# Using a Developmental Approach to Investigate Behavioral, Neurodevelopmental, and Depressive Irritability Types

**DOI:** 10.1016/j.jaacop.2026.02.006

**Published:** 2026-03-04

**Authors:** Aikaterini Bekiropoulou, Olga Eyre, Jon Heron, Kate Langley, Anita Thapar, Lucy Riglin

**Affiliations:** a Wolfson Centre for Young People's Mental Health, Cardiff, United Kingdom; b Cardiff University, Cardiff, United Kingdom; c University of Bristol, Bristol, United Kingdom

**Keywords:** irritability, behavioral, neurodevelopmental, depressive, ALSPAC

## Abstract

**Objective:**

Irritability is highly heterogeneous and a common challenge in youth clinical services. Albeit transdiagnostic, the diagnostic manuals conceptualize severe irritability differently: the *ICD-11* primarily recognizes it as behavioral (oppositional defiant disorder specifier), and the *DSM-5-TR*, as depressive (core to disruptive mood dysregulation disorder). Irritability is also highly prevalent in, and genetically linked to, neurodevelopmental conditions. It is unclear whether irritability represents a unitary construct or multiple different types. We examined whether distinct behavioral, neurodevelopmental, and depressive irritability types, differentiated by their developmental course, sex-preponderance, clinical, genetic, and environmental covariates, could be observed.

**Method:**

Using data from the Avon Longitudinal Study of Parents and Children (female participants: 5,085; male participants: 5,028), we explored sex-stratified irritability latent profiles across 5 time points (∼ages 7-25 years) for irritability measured with the Development and Well-Being Assessment. We investigated associations with various clinical, genetic, and environmental covariates typifying behavioral, neurodevelopmental, and depressive conditions.

**Results:**

We identified 5 irritability profiles similar across sexes (low, child-limited, child/adolescent-limited moderate, child/adolescent-limited high, high-stable) and 2 sex-specific profiles: adolescent-onset (female participants) and fluctuating (male participants). Although most profiles were not distinguished by condition-specific covariates, 2 profiles showed some specificity with neurodevelopmental or depressive conditions: (1) the male high-stable profile was associated with attention-deficit/hyperactivity disorder diagnosis and genetic liability, and autism-like traits, and (2) the female adolescent-onset profile was associated with depression diagnosis and genetic liability, and adolescent/adult stressful life events.

**Conclusion:**

Irritability appears to be developmentally heterogeneous. Albeit often transdiagnostic, for some individuals irritability may align with neurodevelopmental or depressive conditions. This could have potential implications for classification and treatment.

**Study registration information:**

Exploring Possible Behavioural, Neurodevelopmental and Depressive Types of Irritability, Using a Developmental Approach; https://osf.io/av2s8/

## Plain language summary

Irritability is highly impairing and linked to different mental health conditions. Using data from the Avon Longitudinal Study of Parents and Children (n = 10,113), the authors characterized irritability in female and male youths. Five irritability profiles common to female and male youths were identified, as well as adolescent-onset irritability in female youths and fluctuating irritability in male youths. Irritability persisting from childhood to adulthood in male individuals was linked to attention-deficit/hyperactivity disorder and autism, whereas adolescent-onset irritability in female individuals was linked with depression.

Irritability, characterized by developmentally inappropriate proneness to anger and provocation, is highly impairing, and commonly prompts youth clinical referrals.^1^ Conceptualization of severe irritability varies across diagnostic systems^1^: the *International Classification of Diseases 11*^*th*^ *Edition* (*ICD-11*)^2^ includes it as a specifier (dimension) of oppositional defiant disorder (ODD), whereas the *DSM-5-TR*^3^ places it within depressive disorders, specifically as core to disruptive mood dysregulation disorder. In addition, irritability frequently co-occurs in individuals with neurodevelopmental conditions, including autism spectrum disorder (ASD) and attention-deficit/hyperactivity disorder (ADHD).^1^ Irritability is thus highly transdiagnostic. Recent longitudinal work spanning childhood to adolescence indicates that irritability may follow heterogeneous developmental courses, including child-onset persistent or decreasing patterns, and irritability that onsets or increases in adolescence.^4^, ^5^, ^6^, ^7^ There is also evidence to suggest that these courses may also differ meaningfully in their clinical associations, with child-onset persistent irritability aligning closer with neurodevelopmental or behavioral phenotypes, and adolescent-onset irritability perhaps being more depression-like.^4^

We aimed to extend the longitudinal examination of irritability into adulthood, and further examine the hypothesis of different irritability types, distinguished by age-at-onset and persistence. In addition, we aimed to test whether distinct irritability profiles are differentially associated with clinical, genetic, and environmental covariates that typify behavioral, neurodevelopmental, and depressive conditions.

### Clinical and Developmental Heterogeneity in Irritability

Irritability is commonly observed in behavioral disorders and is considered as an ODD dimension.^2^Compared to the other ODD dimensions (headstrong/hurtful), irritability has been found to show similar cross-sectional associations with conduct disorder (CD) symptoms; however, longitudinally, childhood irritability presents weaker associations with adolescent behavioral outcomes.^8^^,^^9^ Support for the behavioral conceptualization of irritability is suggested by findings indicating that it responds well to therapeutic interventions targeting behavioral disorders (eg, parent management training).^10^ Nevertheless, research has indicated considerable heterogeneity in irritability. Longitudinal work has found both childhood and adolescent ODD and CD to be associated more strongly with childhood-onset than with adolescent-onset irritability,^4^ with childhood-onset irritability appearing more commonly in boys than in girls, a pattern also observed in conduct problems.^3^^,^^4^ These findings suggest that childhood-onset irritability, specifically, may be a behavioral-like condition. However, ADHD has also been strongly associated with childhood-onset irritability, making it unclear whether early-onset irritability is better viewed as behavioral or neurodevelopmental. Research has shown that irritability is common in neurodevelopmental conditions, with rates as high as 91% in ADHD cases.^11^ Indeed, emotional dysregulation, a broader concept encompassing irritability, has been recognized as an ADHD-associated feature in the *DSM-5-TR*.^3^ Irritability has also been found to correlate with genetic liability for ADHD in clinical- and population-based cohorts.^12^^,^^13^ If irritability is a feature of a behavioral condition, along with a hypothesized early-onset and male preponderance, we would also expect correlations with core features distinguishing behavioral disorders from other psychopathologies: for example, antisocial behavior, exposure to family conflict, and family history of antisocial behavior.^3^^,^^14^ If a neurodevelopmental irritability profile is observed, we would expect it to present with an early-onset, male preponderance and to display associations with other neurodevelopmental conditions (ASD, cognitive deficits, and special education needs) as well as features characteristic of neurodevelopmental conditions, including perinatal complications.^3^^,^^15^ However, research exploring these behavioral and neurodevelopmental covariates in association with irritability specifically, is limited.

Although irritability often accompanies behavioral and neurodevelopmental conditions, the *DSM-5-TR* and *ICD-11* recognize irritability as a symptom of child/adolescent depression.^3^ Epidemiological evidence also indicates high irritability rates in depressed adults,^16^ suggesting that irritability may represent a depressive-like phenotype in adulthood also. Furthermore, longitudinal research has found the irritable dimension of ODD in childhood to be more predictive of adolescent emotional disorders, such as depression, than the other ODD dimensions.^8^ These findings underscore that irritability for some may have a depressive-like nature. Furthermore, developmental research has further indicated that adolescent-onset irritability correlates with depression genetic liability and, like depression, shows a female preponderance.^4^^,^^17^ Irritability has been also associated with family history of depression,^11^ additionally suggesting shared genetic links. Thus, we hypothesize that depressive irritability would present with an adolescent- or adult-onset (when depression typically commences^17^) and, like depression, would show a female preponderance. It would also be expected to associate with other depression covariates, such as exposure to stressful events^17^; however, again, research exploring these associations is limited.

To summarize, emerging evidence suggests the possibility of distinct irritability types, each presenting with a characteristic developmental course and a broader set of associated features that align with behavioral, neurodevelopmental, and depressive conceptualizations. If distinct irritability types are observed, treatment should address the specific underlying clinical domain; therefore, developmental research clarifying irritability’s clinical heterogeneity is crucial for better classification and treatment protocols. This study aimed to identify distinct developmental profiles of irritability based on irritability onset and persistence. Following identification, we aimed to examine whether these profiles would be differentially associated with clinical, genetic, and environmental covariates characteristic of behavioral, neurodevelopmental, and depressive conditions.

## Method

The study was pre-registered through an Open Science Framework after data collection, but prior to full data access and analysis (link removed for anonymization). Deviations from the protocol are described in the Supplement 1, available online.

### Sample

The Avon Longitudinal Study of Parents and Children (ALSPAC) is a well-established prospective longitudinal birth cohort in the United Kingdom. Pregnant women residing in Avon, UK, with expected delivery dates between April 1, 1991, and December 31, 1992, were invited to participate. Initially, 14,541 pregnancies (14,203 unique mothers) were enrolled; 13,988 children were alive 1 year postpartum. Follow-up recruitment occurred when children reached approximately 7 years, resulting in 15,447 total enrolled pregnancies (14,833 unique mothers; 14,901 children alive 1 year postpartum). Furthermore, 12,113 partners were originally enrolled; currently, 3,807 remain enrolled.^18^ Participants’ data have been collected at multiple ages, through various methods (eg, questionnaires, in-person clinic assessments) and sources (eg, parent-/self-reports). Study data were collected and managed using Research Electronic Data Capture (REDCap) tools hosted at the University of Bristol. REDCap is a secure, Web-based software platform designed to support data capture for research studies.^19^ Additional information on ALSPAC, including assessment waves, can be found elsewhere^20^, ^21^, ^22^; data details are available through the fully searchable data dictionary and variable search tool (https://www.bristol.ac.uk/alspac/researchers/our-data/). We included individuals with any irritability data. In twin pregnancies, we included only the first-born child, except for analyses involving perinatal variables, from which all twins were excluded. Sample demographic characteristics are presented in Table 1.

**Table 1:** Participants’ Demographic Information and Irritability Scores by Age

Variable	Female participants (n = 5,085)		Male participants (n = 5,028)	
	Frequency	%	Frequency	%
**Ethnicity**				
White	4,281	84.2	4,348	86.5
Non-White	189	3.7	202	4
Missing	615	12.1	478	9.5
**Maternal social class by occupation**				
Professional and skilled (non-manual)	3,105	61.1	3,212	63.9
Skilled (manual) and lower	895	17.6	939	18.7
Missing	1,085	21.3	877	17.4
**Paternal social class by occupation**				
Professional and skilled (non-manual)	2,024	39.8	2,039	40.6
Skilled (manual) and lower	1,542	30.3	1,529	30.4
Missing	1,519	29.9	1,460	29
**Maternal highest qualification**				
A level and higher	1,823	35.9	1,846	36.7
O level and lower	2,728	53.6	2,792	55.5
Missing	534	10.5	390	7.8
**Paternal highest qualification**				
A level and higher	2,108	41.5	2,178	43.3
O level and lower	2,311	45.5	2,307	45.9
Missing	666	13.1	541	10.8
**Home ownership status**				
Owned	3,513	69.1	3,648	72.3
Other	870	17.1	783	15.5
Missing	702	13.8	597	12.1
**Irritability**	**Mean**	**SD**	**Mean**	**SD**
7 years	0.42	0.96	0.57	1.20
10 years	0.46	1.02	0.57	1.23
13 years	0.49	1.12	0.45	1.09
15 years	0.58	1.24	0.48	1.13
25 years (parent-reported)	0.36	0.99	0.28	0.91
25 years (self-reported)	1.05	1.43	0.69	1.19

### Measures

#### Irritability

Consistent with previous research,^4^^,^^8^^,^^23^ irritability was measured using the Development And Well-Being Assessment (DAWBA), a validated research diagnostic interview assessing youth mental health disorders.^24^ Parents were asked to complete a questionnaire including 3 ODD items (“severe temper tantrums,” “touchy and easily annoyed,” “angry and resentful”) about their child, at approximately 7, 10, 13, 15, and 25 years of age; at age 25, both parent- and self-ratings of these items were assessed. The individual irritability items were coded as 0 = “no more than others,” 1 = “a little more than others,” and 2 = “a lot more than others”; a summed irritability score (range, 0-6) was then calculated at each age, by combining the 3 items. Although irritability is assessed through other DAWBA dimensions too (eg, post-traumatic stress disorder), these 3 ODD items form the validated irritability construct within the DAWBA,^8^ and align with widely accepted definitions of irritability.^2^^,^^3^ Missingness in the irritability variables by assessment wave is provided in Table S1, available online.

### Behavioral, Neurodevelopmental, and Depressive Covariates

Covariates characterizing the presence of behavioral, neurodevelopmental, and depressive conditions examined are summarized in Table 2 and described in detail below. Behavioral, neurodevelopmental, and depressive domains were not treated as mutually exclusive categories but, rather, as partially overlapping clinical frameworks, each offering a different insight into irritability’s heterogeneity.

**Table 2:** Summary of Behavioral, Neurodevelopmental, and Depressive-Like Variables by Category

Covariate category	Behavioral	Neurodevelopmental	Depressive
**Clinical, cognitive and educational**	Conduct disorder diagnosis (7 and 15 y)	ADHD diagnosis (7, 15, and 25 y)	MDD diagnosis (7, 15, and 25 y)
	Oppositional defiant disorder diagnosis (7 and 15 y)	ASD-like traits (7, 13, and 25 y)	
	Adult antisocial behaviors (25 y)	Special education needs (by 11 y)	
		IQ (8 y)	
**Genetic and family history**	Maternal lifetime antisocial behaviors (child: 12 y)	ADHD PGS	MDD PGS
	Paternal lifetime antisocial behaviors (child: 12 y)	ASD PGS	Maternal lifetime depression (12-wk gestation)
			Paternal lifetime depression (12-wk gestation)
**Environmental**	Parental conflict (child: 6 y)	Preterm birth	Recent independent stressful life events (16 and 25 y)
		Low birth weight	
		Low Apgar score at 5 min	

Note: ADHD = attention-deficit/hyperactivity disorder; ASD = autism spectrum disorder; MDD = major depressive disorder; PGS = polygenic score.

### Clinical, Cognitive, and Educational Covariates

Given the study’s developmental framework, we examined clinical covariates of behavioral, depressive, and neurodevelopmental conditions in childhood, adolescence, and adulthood. Specifically, we used *DSM-IV* diagnoses for ODD (including the 3 irritability items), CD, ADHD, and major depressive disorder (MDD), generated using DAWBA-based computer algorithms that calculate diagnostic probability bands,^24^ whereby >50% probability of diagnosis was coded as meeting diagnostic criteria, at ages 7 and 15 years. In childhood (age 7 years), the parent-rated DAWBA was used for all diagnoses; in adolescence (age 15 years), self-reports were used for MDD and parent-ratings for other diagnoses. At age 25 years, we assessed MDD using the self-rated DAWBA, as previously outlined^25^ and ADHD using the self-rated Barkley Adult ADHD Rating Scale (BAARS-IV) consistent with *DSM-5* criteria, as described elsewhere.^26^ Antisocial behaviors at age 25 were measured using a self-reported 12-item postal questionnaire, based on the offending section of the Edinburgh Study of Youth Transitions and Crime,^27^ assessing the frequency of behaviors such as theft, intentional harm, and property destruction, in the past year. Consistent with prior work, we summed individual item scores to calculate a total score (range, 0-24), with antisocial behaviors coded as “present” if participants’ scores fell within the sample’s top 15%.^28^ ASD-like traits were assessed at ages 7 and 13 years using the parent-rated 12-item Social Communication Disorder Checklist, which measures social communication impairment (range, 0-24; cut-off, ≥9)^29^; previous ALSPAC work has suggested this measure’s strong discriminant validity for child ASD.^30^ At age 25 years, ASD-like traits were evaluated using the self-rated 28-item Autism Spectrum Quotient, which broadly assesses social behaviors and attention to detail (range, 28-112; cut-off, ≥70).^31^ ASD-like traits were coded as “present” for the corresponding time point, if participants’ score met the cut-off. No diagnoses were mutually exclusive.

The presence of special educational needs by age 11 was measured at age 11 years, using teacher-ratings on the “Questionnaire for the Class Teacher”; consistent with prior research,^32^ these were combined into a dichotomous special educational needs “presence”/“absence” variable. IQ was examined using an abbreviated form of the Wechsler Intelligence Scale for Children,^33^ administered in clinic at age 8 years; low IQ was determined by a score of <80.

### Genetic and Family History Covariates

Polygenic scores (PGS) for MDD, ADHD, and ASD were selected because they represent core examples of the conditions typifying our hypotheses. PGS were calculated to assess common variant genetic risk using PRS-CS, which implements a Bayesian regression framework to infer posterior single-nucleotide polymorphisms’ effect sizes, under continuous shrinkage priors^34^ (genetic data quality control available in Supplement 2, available online). Risk alleles were defined using case-control summary statistics from current genome-wide association studies for MDD,^35^ ADHD,^36^ and ASD,^37^ with a linkage disequilibrium reference panel based on the European Ancestry Data from 1000 Genomics Project-Phase 3. PGS were Z score transformed for standardization.

Although PGS for behavioral conditions could in principle be informative, current genome-wide association studies for these remain underpowered or less well validated. Therefore, family history of antisocial behavior was examined for the behavioral component of our hypotheses. These were assessed via self-reports from the child’s biological mother and biological father, each reporting separately their lifetime problems with the law, as answer to “Have you ever been in trouble with the law?” at 12 years (child). Biological maternal and biological paternal lifetime depression was examined using self-reported responses to “Have you ever had severe depression?” which was reported by each biological parent at 12 weeks’ gestation. There were no data available for parental history of neurodevelopmental conditions.

### Environmental Covariates

Parental conflict was measured consistently with previous work,^38^ assessing aspects of parental relationships (arguments, distancing, emotional/physical violence), rated by the mother at age 6 (child). This was coded as “present” if the mother and/or father had engaged in ≥3 conflicting partnership behaviors.^39^ Perinatal complications, including the presence/absence of preterm birth (<37 weeks’ gestation), low birth weight (<2,500 g) and low 5-minute APGAR score (range, 0-10; low, <6), were obtained from obstetric records. Twin pregnancies were excluded because of the higher occurrence of perinatal complications in twins compared to singletons.^40^ Life events previously characterized as independent of one’s control and potentially stressful (eg, family illness/death),^39^ were assessed using the self-rated Recent Life Events checklist, based on established inventories^41^^,^^42^ at ages 16 years (since age 12) and 25 years (since age 24). Antisocial self- and family-related events were excluded, as prior works suggests stronger associations with other conditions, like CD compared to MDD.^43^ This approach ensured that life events assessed remained behavior independent and showed some specificity in associations with depressive rather than behavioral or neurodevelopmental conditions. Total scores (individual items’ score range: 0-4, reflecting events’ presence and perceived impact) were calculated consistent with prior work.^44^

### Data Analyses

Data management and descriptive analyses were conducted in R. We used longitudinal latent profile analysis, including sum scores of parent-rated irritability (ages 7-25 years) and self-rated irritability (age 25 years) separate at each age, to identify latent profiles of irritability using Mplus (8.11); maximum likelihood was used for parameter estimation, and full- information maximum likelihood to address missing data, under the missing-at-random assumption.^45^ Longitudinal latent profile analysis is a data-driven approach that categorizes heterogeneous groups into more homogeneous subgroups, based on mean scores and covariances on the included continuous profile indicators.^45^ Separate models were calculated for male and female participants, as prior work has suggested measurement non-invariance by sex in the parent-rated DAWBA irritability.^46^ Starting with a single profile (k) model, k+1 models were fitted incrementally. Selection of the optimal number of profiles followed recommended parsimony criteria, evaluated by sample size adjusted Bayesian information criterion and bootstrapped likelihood ratio test; entropy and profile size (N ≥ ∼100) were also examined along with theoretical interpretability. Given the lack of strong theoretical rationale suggesting otherwise, variance and covariance were fixed across profiles according to Mplus defaults, to reduce computational demands.^45^

Following profile identification, associations with clinical, genetic, and environmental covariates were assessed as auxiliary information, using the automated Bolck–Croon–Hagenaars (BCH) method, which accounts for measurement error in the latent profile variable when assessing relationships with covariates and allows treatment of binary variables as continuous by calculating the probability of a “1” response.^47^ The profile with the lowest irritability means was used as the reference category. Given the large number of statistical tests performed, we applied false discovery rate correction, specifically the Benjamini–Hochberg procedure, which is appropriate under conditions of positive dependency and therefore consistent with the generally positive correlations among the variables in our sample.^48^ Missing covariate data in the primary analyses were handled with listwise deletion,^45^ with multiple imputation used as sensitivity analysis (Supplement 3, available online).

## Results

Means and frequencies of irritability scores by age, informant, and sex are presented in Table 1 and Figure S1 (available online), respectively. Frequencies of diagnoses and symptoms are displayed in Table S2, available online.

### Irritability Profiles

**Figure 1:**
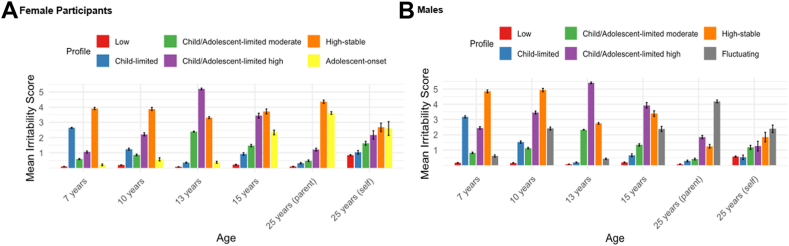
Irritability Scores Across Ages by Profile, Stratified by Sex ***Note:****(A) Female participants. Prevalence by profile: low (80.6%), child-limited (6.1%), child/adolescent-limited moderate (6.1%), child/adolescent-limited high (2.5%), high-stable (1.9%), adolescent-onset (2.9%). (B) Male participants*. *Prevalence by profile: low (80.9%), child-limited (4.9%), child/adolescent-limited moderate (6.2%), child/adolescent-limited high (3%), high-stable (2.3%)****,****fluctuating (2.7%)*

We selected 6-profile solutions for both sexes (Figure 1). These were characterized by different developmental patterns of irritability: 5 profiles were similar across male and female participants, with the sixth profile for each showing a different pattern across sexes. Profiles identified across sexes included the following: low (female participants: 80.6%; male participants: 80.9%), child-limited (female participants: 6.1%; male participants: 4.9%), child/adolescent-limited moderate (female participants: 6.1%; male participants: 6.2%), child/adolescent-limited high (female participants: 2.5%; male participants: 3%), and high-stable (female participants: 1.9%; male participants: 2.3%). Although the child/adolescent-limited profiles generally showed lower irritability levels compared to other elevated irritability profiles in adulthood (eg, the high-stable profile), still adult irritability levels did not match the low profile, particularly in self-reports. The term “limited” therefore reflects this relative attenuation, rather than implying full remission into adulthood. Dissimilarities across sex were as follows: (1) for 2 of these profiles, onset was somewhat earlier for the male participants (child/adolescent-limited profiles were present across childhood and adolescence for the male participants, but only in adolescence for the female participants); and (2) the high-stable profile showed relatively low parent-rated irritability in young adulthood for the male participants, but not for the female participants. The sixth identified profile was an adolescent-onset profile in the female participants (2.9%) and a fluctuating profile in the male participants (2.7%). Details on model selection are available in the Supplement 4, available online (model fit indices: Table S3, cross-tabulation analysis: Table S4, available online); irritability means and standard errors for each profile are displayed in Table S5, available online.

### Covariates

Prevalence rates of clinical, educational and cognitive covariates by profile are shown in Tables 3 and 4, and means and prevalences of genetic and environmental covariates by profile in Tables 5 and 6. Comparisons to the low profile, corrected for multiple testing, are outlined below, separately by profile; original *p* values are given in Tables S6 and S7, available online.

**Table 3:** Prevalence of Clinical, Educational, and Cognitive Covariates by Irritability Latent Profile, and Comparison to Low Profile (Female Participants)

Variables	Low	Child-limited		Child/adolescent-limited (moderate)		Child/adolescent- limited (high)		High-stable		Adolescent-onset	
	Prevalence (SE)	Prevalence (SE)	χ^2^ (*q*)^a^	Prevalence (SE)	χ^2^ (*q*)	Prevalence (SE)	χ^2^ (*q*)	Prevalence (SE)	χ^2^ (*q*)	Prevalence (SE)	χ^2^ (*q*)
**Behavioral**											
Oppositional defiant disorder 7 y	–	**14.4%** (2.2%)	43.287 (0.007)	–	0.500 (0.611)	4.7% (3%)	2.686 (0.180)	**36.7%** (6.1%)	36.436 (0.007)	–	0.170 (0.776)
Oppositional defiant disorder 15 y	–	2.1% (1.5%)	3.050 (0.155)	**8.5%** (2.2%)	15.951 (0.007)	**48.6%** (8.9%)	30.469 (0.007)	**42.5%** (7.6%)	31.932 (0.007)	**18.2%** (5.3%)	11.924 (0.007)
Conduct disorder 7 y	–	**2.6%** (1%)	6.749 (0.028)	–	0.906 (0.458)	–	0.471 (0.616)	7.8% (3.4%)	5.298 (0.053)	–	5.803 (0.041)
Conduct disorder 15 y	–	–	0.945 (0.449)	**4.3%** (1.6%)	7.290 (0.025)	**18.4%** (6.5%)	8.076 (0.018)	**13.7%** (5.3%)	6.707 (0.029)	**11.1%** (4.2%)	6.854 (0.028)
Antisocial behaviors 25 y	9.3% (0.7%)	9.5% (2.6%)	0.011 (0.935)	**17.7%** (3.3%)	6.085 (0.036)	23.8% (7.1%)	4.111 (0.090)	**29.7%** (7.8%)	6.858 (0.028)	**26.6%** (5.7%)	8.555 (0.014)
**Neur-odevelopmental**											
ADHD 7 y	0.2% (0.1%)	**3.3%** (1.1%)	7.300 (0.025)	–	0.109 (0.820)	–	0.083 (0.839)	**13.6%** (4.3%)	9.843 (0.012)	–	0.051 (0.869)
ADHD 15 y	–	–	1.808 (0.276)	–	0.214 (0.741)	4% (3.5%)	1.372 (0.342)	**15.9%** (5.6%)	8.227 (0.018)	5.5% (3%)	3.170 (0.145)
ADHD 25 y	2.1% (0.4%)	3.5% (1.6%)	0.703 (0.520)	1.8% (1.1%)	0.081 (0.839)	5.3% (3.9%)	0.665 (0.533)	**20.9%** (6.7%)	7.844 (0.020)	10.4% (3.9%)	4.209 (0.085)
ASD-like traits 7 y	1.7% (0.3%)	**22.8%** (2.7%)	57.665 (0.007)	5% (1.5%)	4.626 (0.070)	**16.8%** (4.8%)	9.746 (0.012)	**52.6%** (6.6%)	60.047 (0.007)	–	0.060 (0.867)
ASD-like traits 13 y	3.5% (0.5%)	**11.8%** (2.5%)	9.991 (0.012)	**19.4%** (2.8%)	28.815 (0.007)	**67.2%** (8.9%)	51.503 (0.007)	**58.6%** (7.9%)	48.287 (0.007)	**30.9%** (6.6%)	16.542 (0.007)
ASD-like traits 25 y	10.4% (0.7%)	14.2% (3.1%)	1.398 (0.340)	11.5% (2.7%)	0.144 (0.792)	**40.6%** (8.3%)	12.979 (0.007)	**42.1%** (8.3%)	14.386 (0.007)	**27.9%** (5.8%)	8.583 (0.014)
Low IQ	4.7% (0.5%)	5.8% (1.7%)	0.373 (0.659)	8.9% (1.9%)	4.406 (0.079)	13.1% (4.5%)	3.350 (0.131)	**19.6%** (5%)	8.867 (0.014)	11.4% (4.3%)	2.274 (0.208)
Special education needs	13.3% (0.9%)	19.8% (3.9%)	2.667 (0.180)	16.1% (3.2%)	0.726 (0.515)	**36.8%** (7.9%)	8.714 (0.014)	**32.1%** (7.3%)	6.523 (0.031)	22.2% (7.2%)	1.472 (0.328)
**Depressive**											
Major depressive disorder 7 y	–	**4.8%** (1.3%)	12.356 (0.007)	–	0.439 (0.624)	–	12.668 (0.007)	–	1.726 (0.285)	–	6.276 (0.032)
Major depressive disorder 15 y	1.9% (0.3%)	4.6% (1.7%)	2.532 (0.195)	2.5% (1.2%)	0.302 (0.697)	–	0.102 (0.824)	5.8% (3.3%)	1.364 (0.342)	–	0.017 (0.921)
Major depressive disorder 25 y	8.2% (0.7%)	9.8% (2.6%)	0.325 (0.688)	15.5% (3%)	5.189 (0.055)	**29.4%** (7.4%)	7.980 (0.020)	**27.4%** (7.3%)	6.805 (0.028)	**38.2%** (6.2%)	21.781 (0.007)

Note: Cells marked with a dash (–) indicate n < 5; this may include 0%. χ^2^ Compared to low profile (df = 1). ADHD = attention-deficit/hyperactivity disorder; ASD = autism spectrum disorder.

a *q* Values adjusted for multiple testing (statistically significant values are in boldface type); unadjusted *p* values are shown in Table S6, available online.

**Table 4:** Prevalence of Clinical, Educational, and Cognitive Covariates by Irritability Latent Profile and Comparison to Low Profile (Male Participants)

Variables	Low	Child-limited		Child/adolescent- limited (moderate)		Child/ adolescent- limited (high)		High-stable		Fluctuating	
	Prevalence (SE)	Prevalence (SE)	χ^2^ (*q*)^a^	Prevalence (SE)	χ^2^ (*q*)	Prevalence (SE)	χ^2^ (*q*)	Prevalence (SE)	χ^2^ (*q*)	Prevalence (SE)	χ^2^ (*q*)
**Behavioral**											
Oppositional defiant disorder 7 y	–	**31%** (3.5%)	74.692 (0.005)	**4.8%** (1.4%)	10.471 (0.005)	**24.4%** (4.8%)	25.365 (0.005)	**80.4%** (4.8%)	285.192 (0.005)	4.5% (2.9%)	2.408 (0.231)
Oppositional defiant disorder 15 y	–	–	0.173 (0.745)	**12.4%** (2.7%)	19.489 (0.005)	**89.4%** (18.9%)	22.449 (0.005)	**54.5%** (8.9%)	37.661 (0.005)	**27.4%** (6.6%)	16.912 (0.005)
Conduct disorder 7 y	–	3.4% (1.5%)	5.265 (0.063)	–	1.231 (0.412)	**11.5%** (3.4%)	11.534 (0.005)	**15.7%** (4.1%)	14.631 (0.005)	–	0.075 (0.824)
Conduct disorder 15 y	–	–	0.024 (0.901)	3% (1.4%)	3.898 (0.118)	**33.4%** (13%)	6.619 (0.035)	11.3% (5.6%)	4.018 (0.112)	**15.1%** (5.2%)	8.254 (0.016)
Antisocial behaviors 25 y	15.8% (1.2%)	13.3% (5.2%)	0.228 (0.712)	**31.8%** (5.6%)	7.648 (0.022)	27% (23.9%)	0.219 (0.714)	35.7% (12.4%)	2.570 (0.211)	**37.6%** (8.9%)	5.837 (0.049)
**Neur-odevelopmental**											
ADHD 7 y	–	**13.2%** (2.6%)	23.029 (0.005)	**4.3%** (1.3%)	7.854 (0.020)	**23%** (4.6%)	23.837 (0.005)	**33.4%** (5.2%)	39.692 (0.005)	8.1% (3.2%)	5.591 (0.053)
ADHD 15 y	–	3.3% (2.1%)	2.158 (0.253)	1.8% (1.1%)	2.007 (0.271)	**33.7%** (13%)	6.640 (0.035)	14.3% (6.1%)	5.299 (0.061)	7.1% (3.8%)	3.319 (0.149)
ADHD 25 y	2% (0.4%)	–	0.417 (0.612)	4.9% (2.5%)	1.197 (0.418)	19.4% (17%)	1.039 (0.451)	23.2% (10.4%)	4.112 (0.109)	12.5% (6.2%)	2.809 (0.184)
ASD-like traits 7 y	2.2% (0.4%)	**52.1%** (4%)	152.209 (0.005)	**20.7%** (2.7%)	45.618 (0.005)	**42.8%** (5.8%)	48.894 (0.005)	**68.7%** (5.4%)	152.782 (0.005)	**16.6%** (4.7%)	9.332 (0.008)
ASD-like traits 13 y	3.3% (0.5%)	**21.3%** (4.4%)	15.948 (0.005)	**23.3%** (3.2%)	37.294 (0.005)	**98.3%** (16.1%)	34.951 (0.005)	**48.1%** (8.7%)	26.424 (0.005)	**21.6%** (6%)	9.282 (0.008)
ASD-like traits 25 y	18.1% (1.2%)	12.7% (5.4%)	0.925 (0.473)	19.7% (5%)	0.101 (0.800)	42.7% (24.9%)	0.982 (0.466)	39.8% (12.2%)	3.157 (0.157)	33.5% (8.9%)	2.912 (0.175)
Low IQ	5.9% (0.5%)	8.1% (2.6%)	0.743 (0.528)	8.6% (1.9%)	1.975 (0.273)	**36.4%** (7.5%)	16.747 (0.005)	15.9% (4.7%)	4.563 (0.087)	8.7% (3.6%)	0.614 (0.566)
Special education needs	27.8% (1.1%)	**48.8%** (5.7%)	12.821 (0.005)	29.5% (3.8%)	0.172 (0.745)	**72%** (8%)	30.136 (0.005)	**60.3%** (7.6%)	17.870 (0.005)	38.9% (7.4%)	2.141 (0.253)
**Depressive**											
Major depressive disorder 7 y	–	**3.8%** (1.5%)	6.222 (0.042)	–	1.162 (0.424)	4.5% (2.3%)	3.777 (0.124)	**12%** (3.6%)	10.835 (0.005)	–	17.433 (0.005)
Major depressive disorder 15 y	0.5% (0.2%)	–	0.001 (0.981)	–	0.404 (0.614)	11.5% (7.1%)	2.372 (0.234)	5.1% (3.7%)	1.548 (0.341)	4.9% (2.9%)	2.166 (0.253)
Major depressive disorder 25 y	4.7% (0.7%)	7.9% (4%)	0.624 (0.566)	6.3% (3%)	0.258 (0.693)	–	0.040 (0.872)	11.4% (7.9%)	0.717 (0.528)	**30.1%** (8.3%)	9.180 (0.008)

Note: Cells marked with a dash (–) indicate n < 5; this may include 0%. χ^2^ Compared to low profile (df=1). ADHD = attention-deficit/hyperactivity disorder; ASD = autism spectrum disorder.

a *q* Values adjusted for multiple testing (statistically significant values are in boldface type); unadjusted *p* values are shown in Table S6, available online.

**Table 5:** Means and Prevalence of Genetic, Family History, and Environmental Covariates by Irritability Latent Profile and Comparison to Low Profile (Female Participants)

Variables	Low	Child-limited		Child/ adolescent- limited (moderate)		Child/adolescent- limited (high)		High-stable		Adolescent-onset	
	Prevalence/mean (SE)	Prevalence/mean (SE)	χ^2^ (*q*)^a^	Prevalence/mean (SE)	χ^2^ (*q*)	Prevalence/mean (SE)	χ^2^ (*q*)	Prevalence/mean (SE)	χ^2^ (*q*)	Prevalence/mean (SE)	χ^2^ (*q*)
**Behavioral**											
Maternal Antisocial Behaviors	7.1% (0.6%)	**14.1%** (2.5%)	7.190 (0.025)	10.4% (2%)	2.421 (0.200)	**21.9%** (5.7%)	6.567 (0.029)	**19.1%** (5.4%)	4.897 (0.063)	8.6% (3.9%)	0.138 (0.792)
Paternal antisocial Behaviors	28.4% (1.4%)	35.5% (5.2%)	1.676 (0.291)	38.4% (5%)	3.504 (0.121)	60.1% (20.9%)	2.294 (0.207)	21.9% (9.4%)	0.473 (0.616)	39.3% (8.4%)	1.555 (0.314)
Parental Conflict	9.1% (0.6%)	14.6% (2.4%)	4.693 (0.069)	**15.9%** (2.5%)	6.662 (0.029)	**24.5%** (5.9%)	6.822 (0.028)	**26.3%** (6.1%)	7.976 (0.020)	19.9% (5.5%)	3.710 (0.112)
**Neuro-developmental**											
ADHD PGS^b^	−0.076 (0.020)	−0.002 (0.076)	0.869 (0.467)	**0.080** (0.071)	4.271 (0.084)	**0.337** (0.150)	7.398 (0.025)	0.157 (0.149)	2.409 (0.200)	**0.333** (0.128)	9.648 (0.012)
ASD PGS^b^	−0.027 (0.021)	0.060 (0.076)	1.178 (0.384)	0.096 (0.069)	2.834 (0.171)	0.145 (0.127)	1.792 (0.276)	0.211 (0.153)	2.392 (0.200)	0.195 (0.143)	2.295 (0.207)
cPreterm birth	4.5% (0.4%)	2.6% (1%)	2.742 (0.179)	3.2% (1.1%)	1.222 (0.375)	–	0.288 (0.697)	5.9% (2.6%)	0.290 (0.697)	4.6% (2.3%)	0.002 (0.972)
Low birth weight	3.4% (0.3%)	3.4% (1.2%)	0.002 (0.972)	5.8% (1.4%)	2.387 (0.200)	4.3% (2.3%)	0.144 (0.792)	–	0.000 (0.990)	4.7% (2.4%)	0.252 (0.720)
Low Apgar score	0.4% (0.1%)	–	0.056 (0.868)	–	0.047 (0.870)	–	0.457 (0.618)	–	9.140 (0.014)	–	10.108 (0.007)
**Depressive**											
Major depressive disorder PGS^b^	−0.092 (0.020)	0.033 (0.072)	2.726 (0.179)	**0.100** (0.066)	7.259 (0.025)	**0.266** (0.133)	7.059 (0.028)	0.190 (0.133)	4.384 (0.079)	**0.673** (0.150)	24.525 (0.007)
Maternal depression	6% (0.4%)	**11.3%** (2%)	6.401 (0.031)	**12.7%** (2.1%)	9.551 (0.012)	**16.2%** (4.1%)	6.007 (0.036)	9.6% (3.4%)	1.056 (0.416)	15.6% (4.1%)	5.276 (0.053)
Paternal depression	5.8% (0.5%)	6% (1.8%)	0.018 (0.921)	6.7% (1.9%)	0.222 (0.739)	13.8% (5%)	2.517 (0.195)	–	3.688 (0.112)	3.5% (2.5%)	0.768 (0.502)
Stressful life events (16 y)	5.74 (0.106)	6.319 (0.281)	3.573 (0.119)	6.299 (0.376)	1.958 (0.253)	**9.469** (1.127)	10.818 (0.007)	7.052 (0.844)	2.381 (0.200)	**7.732** (0.769)	6.317 (0.032)
Stressful life events (25 y)^b^	5.911 (0.118)	5.821 (0.453)	0.036 (0.877)	7.205 (0.540)	5.270 (0.053)	5.113 (0.647)	1.467 (0.328)	7.534 (0.956)	2.839 (0.171)	**8.493** (0.857)	8.532 (0.014)

Note: Cells marked with a dash (–) indicate n < 5; this may include 0%. χ^2^ Tests compared to low profile as reference (df = 1). ADHD = attention-deficit/hyperactivity disorder; ASD = autism spectrum disorder; PGS = polygenic score.

a *q* Values adjusted for multiple testing (statistically significant values are in boldface type); unadjusted *p* values are shown in Table S7, available online.

b Mean.

**Table 6:** Means and Prevalence of Genetic, Family History, and Environmental Covariates by Irritability Latent Profile and Comparison to Low Profile (Male Participants)

Variables	Low	Child-limited		Child/ adolescent- limited (moderate)		Child/adolescent- limited (high)		High-stable		Fluctuating	
	Prevalence/mean (SE)	Prevalence/mean (SE)	χ^2^ (*q*)^a^	Prevalence/mean (SE)	χ^2^ (*q*)	Prevalence/mean (SE)	χ^2^ (*q*)	Prevalence/mean (SE)	χ^2^ (*q*)	Prevalence/mean (SE)	χ^2^ (*q*)
**Behavioral**											
Maternal antisocial behaviors	6.6% (0.5%)	12.2% (2.9%)	3.424 (0.145)	8.3% (1.9%)	0.723 (0.528)	21.3% (7.1%)	4.321 (0.098)	14.8% (4.5%)	3.259 (0.154)	15.4% (4.5%)	3.619 (0.133)
Paternal antisocial behaviors	28% (1.3%)	27.2% (5.9%)	0.019 (0.901)	34.4% (4.9%)	1.546 (0.341)	60% (17.3%)	3.389 (0.147)	21.6% (9.6%)	0.445 (0.610)	37.3% (12.8%)	0.506 (0.601)
Parental conflict	8.7% (0.5%)	12.6% (2.9%)	1.703 (0.313)	11.7% (2.2%)	1.812 (0.297)	17.2% (4.8%)	3.211 (0.156)	15.3% (4.3%)	2.322 (0.238)	**20.1%** (4.5%)	6.180 (0.042)
**Neuro-developmental**											
ADHD PGS^b^	−0.085 (0.019)	−0.001 (0.085)	0.887 (0.482)	0.059 (0.069)	3.855 (0.121)	0.189 (0.154)	3.090 (0.159)	**0.259** (0.102)	11.017 (0.005)	**0.250** (0.105)	9.642 (0.008)
ASD PGS^b^	0.001 (0.019)	0.061 (0.089)	0.428 (0.612)	−0.059 (0.065)	0.760 (0.528)	0.094 (0.142)	0.416 (0.612)	0.036 (0.120)	0.086 (0.814)	0.080 (0.111)	0.477 (0.607)
Preterm birth	4.7% (0.4%)	7.4% (2%)	1.717 (0.313)	5% (1.3%)	0.047 (0.865)	6.3% (2.6%)	0.341 (0.644)	10% (3%)	3.176 (0.157)	6.9% (2.5%)	0.736 (0.528)
Low birth weight	3.5% (0.3%)	5.3% (1.7%)	1.072 (0.445)	2.7% (1%)	0.563 (0.586)	5.3% (2.4%)	0.487 (0.606)	6.6% (2.5%)	1.502 (0.347)	3.8% (1.9%)	0.020 (0.901)
Low Apgar score	–	–	0.448 (0.610)	–	0.008 (0.937)	–	0.106 (0.800)	–	2.215 (0.251)	–	16.574 (0.005)
**Depressive**											
Major depressive disorder PGS^b^	−0.082 (0.019)	−0.020 (0.080)	0.550 (0.588)	0.052 (0.072)	3.132 (0.157)	0.249 (0.146)	4.995 (0.068)	**0.224** (0.110)	7.451 (0.022)	**0.280** (0.113)	9.780 (0.008)_
Maternal depression	6.6% (0.4%)	**13.5%** (2.7%)	6.301 (0.040)	9.2% (1.8%)	1.939 (0.277)	**20.2%** (4.2%)	10.210 (0.005)	**15.8%** (3.7%)	6.095 (0.044)	5.8% (2.6%)	0.102 (0.800)
Paternal depression	4.4% (0.4%)	10.4% (2.7%)	4.559 (0.087)	5.3% (1.6%)	0.291 (0.672)	8.2% (3.9%)	0.957 (0.466)	13.9% (4%)	5.655 (0.051)	6.3% (3.3%)	0.349 (0.644)
Stressful life events (16 y)^b^	4.235 (0.096)	4.761 (0.489)	1.086 (0.444)	**5.436** (0.442)	6.830 (0.033)	7.260 (2.622)	1.329 (0.388)	6.436 (0.975)	5.052 (0.068)	4.926 (1.008)	0.461 (0.610)
Stressful life events (25 y)^b^	4.931 (0.145)	4.386 (0.527)	0.966 (0.466)	6.071 (0.582)	3.513 (0.140)	5.898 (2.680)	0.130 (0.784)	4.139 (1.105)	0.505 (0.601)	6.356 (0.976)	2.048 (0.266)

Note: Cells marked with a dash (–) indicate n < 5; this may include 0%. χ^2^ Tests compared to low profile as reference (df = 1). ADHD = attention-deficit/hyperactivity disorder; ASD = autism spectrum disorder; PGS = polygenic score.

a *q* Values adjusted for multiple testing (statistically significant values are in boldface type); unadjusted *p* values are shown in Table S7, available online.

b Mean.

### Child-Limited Profile

Compared to the low profile (Tables 3 and 4), the child-limited profile showed higher prevalence across childhood conditions, which, in this profile, ranged from 2.6% to 31% (behavioral), 3.3% to 52.1% (neurodevelopmental), and 3.8% to 4.8% (depressive) across sexes. In male participants, this profile was also associated with higher rates of special education needs (48.8%). For the genetic, family history, and environmental covariates (Table 5), compared to the low profile, this profile showed association with increased rates of maternal antisocial behavior in female participants. No strong evidence of association with neurodevelopmental genetic or environmental covariates was observed for either sex (Tables 5 and 6). Finally, this profile showed higher rates of maternal depression in both sexes, compared to the low profile (Tables 5 and 6).

### Child/Adolescent-Limited Profile (Moderate and High Profiles)

In the female participants, relative to the low profile (Table 3), the child/adolescent-limited moderate and child/adolescent-limited high profiles were associated with higher levels of behavioral conditions (ODD and CD), which in adolescence ranged from 4.3% to 8.5% and 18.4% to 48.6%, respectively, and in adulthood were 17.7% for the moderate profile. These profiles also showed higher levels of ASD-like traits in childhood (high profile: 16.8%), adolescence (19.4% and 67.2% respectively) and adulthood (high profile: 40.6%). The high profile was also associated with adult MDD (29.4%) and special education needs (36.8%) (Table 3). In terms of genetic, family history, and environmental covariates (Table 5), relative to the low profile, maternal antisocial behaviors and adolescent stressful life events were elevated in the child/adolescent-limited high profile; parental conflict, ADHD, and MDD PGS, as well as maternal depression were elevated in both profiles.

In male participants, compared to the low profile, these profiles were associated with elevated rates of both behavioral (ODD, and for the high-profile CD) and neurodevelopmental conditions (ADHD and ASD-like traits) in childhood and adolescence; adult antisocial behaviors were elevated in the moderate profile (Table 4). The high profile also showed increased rates of low IQ (36.4%) and special education needs (72%) compared to the low profile (Table 4). There was not strong evidence of association with MDD for either male profile. Regarding genetic, family history, and environmental covariates, compared to the low profile (Table 6), there was evidence of higher rates of maternal depression in the high profile and of adolescent stressful events in the moderate profile.

### High-Stable Profile

Relative to the low profile (Tables 3 and 4), the high-stable profiles had increased prevalence across most conditions at most ages. Behavioral disorders in the female high-stable profile were 36.7% in childhood, 13.7% to 42.5% in adolescence, and 29.7% in adulthood, and in the equivalent male profile, behavioral conditions were elevated only in childhood, ranging from 15.7% to 80.4%, and in adolescence (54.5%). Neurodevelopmental conditions in this profile were also higher compared to those in the low profile (Tables 3 and 4) for both sexes in childhood (female participants: 13.6%-52.6%; male participants: 33.4%-68.7%), adolescence (female participants: 15.9%-58.6%; male participants: 48.1%), and in female participants also in adulthood (20.9%-42.1%). Special education needs (female participants: 32.1%; male participants: 60.3%) and low IQ (female participants: 19.6%) were likewise elevated in the high-stable profiles. MDD rates in this profile were high in adulthood for female participants (27.4%) and in childhood for male participants (12%), compared to those in the low profile (Tables 3 and 4). Regarding genetic, family history, and environmental covariates, relative to the low profile, maternal antisocial behaviors and family conflict were associated with the female profile, whereas in the male profile, associations were observed with ADHD and MDD PGS, and maternal depression (Tables 5 and 6).

### Adolescent-Onset Profile (Female Participants Only)

Relative to the low profile (Table 3), the adolescent-onset profile was associated with increased rates of behavioral conditions and ASD-like traits in adolescence (11.1%-18.2% and 30.9%, respectively) and adulthood (26.6% and 27.9%, respectively), as well as MDD in adulthood (38.2%). For the genetic, family history, and environmental covariates (Table 5), there was not strong evidence of association with behavioral variables; conversely, associations were found for ADHD and MDD PGS, and stressful life events in adolescence and adulthood.

### Fluctuating Profile (Male Participants Only)

Compared to the low profile, the fluctuating profile was associated with heightened prevalence of behavioral conditions, which, in this profile, ranged from 15.1% to 27.4% in adolescence and were 37.6% in adulthood, ASD-like traits in childhood (16.6%) and adolescence (21.6%) and MDD in adulthood (30.1%) (Table 4). In terms of genetic, family history, and environmental covariates (Table 6), this profile showed higher rates than did the low profile for parental conflict, as well as ADHD and MDD PGS.

### Post Hoc Analyses: Polygenic Scores

Where a profile showed elevated levels of multiple PGS in univariable analyses (Tables 5 and 6), we performed post hoc multivariable logistic regression analyses, using the R3STEP method.^45^ In female participants, this applied to the child/adolescent-limited, high-stable and adolescent-onset profiles, and in male participants, to the high-stable and fluctuating profiles–all of which showed evidence of association with ADHD PGS and MDD PGS in univariable analyses. For the female child/adolescent-limited moderate profile, multivariate analyses indicated association with MDD PGS (odds ratio [OR = 1.18; 95% CI = 1.01-1.37) but did not find strong evidence for ADHD PGS (OR = 1.11; 95% CI = 0.60-1.29); the opposite was observed for the female child/adolescent-limited high profile (ADHD PGS OR = 1.42; 95% CI = 1.07-1.90; MDD PGS OR = 1.31; 95% CI = 0.99-1.74). The female adolescent-onset profile showed association with MDD PGS (OR = 2.19; 95% CI = 1.59-3.00), but we did not find strong evidence for ADHD PGS (OR = 1.21; 95% CI = 0.91-1.60). The male high-stable profile was associated with ADHD PGS (OR = 1.30; 95% CI = 1.04-1.69), but there was no strong evidence for MDD PGS (OR = 1.25; 95% CI = 0.99-1.59). In the fluctuating profile, both ADHD PGS (OR = 1.28; 95% CI = 1.00-1.65) and MDD PGS (OR = 1.34; 95% CI = 1.04-1.71) showed association in multivariable analyses.

### Post Hoc Analyses: Differentiating Profiles

As primary analyses indicated that the male high-stable profile may be neurodevelopmental-like and the female adolescent-onset profile may be depressive-like, we conducted post hoc sex-specific comparisons between these profiles and the 4 other irritability profiles on neurodevelopmental and depressive covariates, respectively. Male participants in the high-stable profile showed the highest prevalence of ADHD in childhood and ASD-like traits in childhood and adolescence (Table 4), although rates of ADHD were similar for the high-stable and child/adolescent-limited high profiles. In adulthood, there was no strong evidence that rates were higher for either condition in the high-stable compared to other irritability profiles (Table S8, available online). Furthermore, although the high-stable profile showed the highest levels of neurodevelopmental genetic and perinatal complications (Table 6) and among the highest for educational and cognitive covariates (Table 4), statistical evidence of differences across profiles was weak (Table S8, available online).

The female adolescent-onset profile showed the highest rates of adult MDD (38.2%, vs 8.2-29.4% in other profiles) and the highest MDD PGS and number of adult stressful life events, although statistical evidence of differences across profiles was variable (Table S9, available online).

### Sensitivity Analyses

Sensitivity analyses conducted using multiple imputation to address missing covariate data (imputed based on variables presented in Table S10, available online) showed broadly similar patterns with the non-imputed data (Tables S11 and S12, available online), with somewhat higher prevalence rates of clinical, educational, and cognitive covariates (Table S11, available online).

## Discussion

We identified diverse irritability latent profiles from childhood to young adulthood, building on prior longitudinal work that has shown similarly varied developmental courses from childhood into adolescence, and extending these patterns from childhood into adulthood.^4^, ^5^, ^6^, ^7^ Five profiles were similar across sexes (onsetting slightly earlier in male participants), and 2 profiles were sex specific. Apart from the low profile, all others were characterized by elevated irritability as follows: (1) limited to childhood and/or adolescence; (2) emerging in adolescence and persisting into adulthood; or (3) prevalent across childhood, adolescence, and young adulthood (stable or fluctuating course). Most profiles with heightened irritability also exhibited relatively high rates of behavioral, neurodevelopmental, and depressive conditions and/or covariates. Nonetheless, 2 profiles appeared to align more closely with the developmental course, sex preponderance, and phenotypic/genetic characteristics of specific conditions: (1) the male high-stable profile with neurodevelopmental conditions, and (2) the female adolescent-onset profile, with depression. These sex differences should, however, be interpreted considering potential methodological influences, including sex differences in help seeking, symptom reporting, and possible rater bias in parent-reported measures. Finally, although behavioral covariates were common across profiles, no profile appeared behavioral only without underlying neurodevelopmental or depressive comorbidity.

Although neurodevelopmental characteristics were observed across profiles with elevated irritability, the high-stable profile in male participants was most consistently associated with neurodevelopmental conditions. Irritability is not officially recognized as core to neurodevelopmental conditions’ diagnostic criteria; however, it is commonly prevalent among neurodivergent individuals.^1^ As hypothesized, this profile, which aligned with neurodevelopmental conditions, was observed somewhat more commonly in male (2.3%) than in female (1.9%) participants, and was marked by early-onset irritability of stable course from childhood into adulthood, alongside elevated rates of ADHD and ASD-like traits.^3^^,^^15^ This profile also shared genetic vulnerability for ADHD (PGS), consistent with previous work on childhood-onset irritability.^13^ Comorbidity rates, particularly for ODD, were also notably high, consistent with work suggesting high comorbidity between neurodevelopmental and behavioral conditions.^15^ An unexpected finding regarding this profile was the high prevalence of childhood MDD. Although irritability may increase depression risk in individuals with ADHD,^11^ the limited evidence of association with MDD PGS, and the atypically early onset, may suggest that mechanisms underlying this childhood MDD could be distinct from those in “typical” MDD, potentially aligning with previous work suggesting that early-onset depression may be neurodevelopmental in nature.^49^ Overall, our findings are consistent with previous research suggesting that early-onset persistent irritability, particularly in male individuals, may align with neurodevelopmental conditions.^4^ However, this profile also showed some of the highest prevalences of ODD, CD, and childhood MDD, indicating a broad pattern of clinical involvement.

In line with our hypothesis, we also identified a female pattern of irritability onsetting in adolescence and appearing to show comparatively stronger depressive involvement than other profiles, including adult MDD and depression risk factors (adolescent and adult behavior-independent stressful life events, maternal depression, and MDD PGS). Although stressful experiences may contribute to multiple psychiatric conditions inside and outside the scope of our work,^3^^,^^43^ our focus on behavior-independent events is important, as evidence suggests that uncontrollable/non–self-generated events may be more consistently associated with depressive conditions, thus showing clearer differentiation from behavioral or neurodevelopmental pathways.^4^^,^^23^^,^^43^ Interestingly, this group had elevated rates of ASD-like traits in adolescence and adulthood but not in childhood, perhaps presenting with a “late-onset” neurodevelopmental condition.^50^ Moreover, the adolescent-onset profile also showed relatively elevated rates of adult antisocial behaviors, consistent with prior research that suggests that irritability in depressive presentations can increase susceptibility to disruptive behavior, especially among female participants.^51^ Overall, these findings are consistent with previous work, suggesting that adolescent-onset irritability in female individuals may be depressive in nature.^4^ Our results support this, suggesting that for this profile, adolescent-onset irritability may persist into adulthood and be accompanied by additional depressive features, although depressive features may not be unique to this profile. Although irritability is currently recognized as a symptom of child or adolescent depression,^3^ our findings indicate that for some individuals, irritability may also be related to adult depression.

Regarding our final hypothesis of a behavioral irritability type, rates of behavioral conditions (ODD, CD, antisocial behaviors) were elevated across irritability profiles, consistent with its prominence in disruptive behavior disorders (indeed, being an ODD specifier in diagnostic manuals^2^). Profiles with particularly high rates of behavioral features, including CD and antisocial behaviors, were those with a high level of irritability (child/adolescent-limited high, high-stable). However, contrary to our hypotheses, neither appeared distinctly behavioral; neurodevelopmental or depressive characteristics were also present, particularly in the high-stable profile in female participants, which showed some of the highest rates of these features. A potential explanation for this could be that irritability was measured using ODD items, which may have introduced a behavioral element across profiles. The child/adolescent-limited (high) profile and the high-stable profile in female participants presented with a range of behavioral, neurodevelopmental, and depressive conditions, particularly in adolescence and adulthood, as well as notably high levels of special education needs and low IQ. Thus, although irritability is classified as a behavioral disorder specifier within ODD in the *ICD-11,*^2^ our findings did not yield strong evidence for a distinctly behavioral profile; although irritability may often co-occur with behavioral conditions, this appears often to be in the context of other conditions.

Interestingly, we also identified a fluctuating profile in male participants, with somewhat episodic irritability in childhood and adolescence, and high levels of adult irritability—a pattern most pronounced in parent-ratings, albeit highest across both parent- and self-ratings compared to the other profiles. A possible explanation for this informant discrepancy could be that parents may be more attuned to outward expressions of irritability that may arise in family interactions. Conversely, self-ratings may reflect internal symptom appraisal, which may differ from expressions apparent to external observers such as parents. In addition, it is possible that heightened irritability in this profile may arise mainly within family interactions but may be less severe in other settings, contributing to an overall lower self-reported symptom severity. More broadly, incorporating multiple informants could be valuable, as individuals with some conditions may under-report their own symptoms; for instance, research has shown that people with ADHD often under-estimate or under-report their depressive symptoms.^52^ Although an episodic pattern might be expected to align with depressive phenotypes, this profile was associated with different types of conditions at different developmental stages: most notably, neurodevelopmental in childhood, behavioral in adolescence, and depressive in adulthood. It was also associated with family conflict and ADHD genetic liability. This developmental variation is consistent with heterotypic continuity, possibly because of shared risk factors or the presence of one condition increasing the risk of developing another.^53^ Potentially, in this profile of fluctuating irritability, irritability serves as a developmental “bridge” between neurodevelopmental and depressive conditions.

Finally, we found a child-limited irritability profile (across sexes), which showed elevated behavioral, neurodevelopmental, and depressive conditions in childhood; beyond childhood, this profile largely resembled the low irritability profile. This pattern appears consistent with evidence suggesting that irritability could be common in childhood and typically decrease over time,^1^ possibly because of developmental maturation in areas such as executive function and social–communication skills.^54^

Overall, to our knowledge, this research is the first to investigate the developmental course of irritability in a large longitudinal cohort across childhood, adolescence, and adulthood. Testing the hypothesis of behavioral, neurodevelopmental, and depressive irritability types, we found that, although irritability for most individuals appeared to be largely transdiagnostic, clinically distinct irritability profiles that seem to align with neurodevelopmental and depressive phenotypes, also emerged. This suggests that the developmental context of irritability may be important for classification and treatment. The age-of-onset and persistence of irritability is of particular relevance: for example, if early-onset persistent irritability is neurodevelopmental in nature, it may benefit from therapeutic approaches typically targeting neurodevelopmental domains; conversely, if later-onset irritability is a depression-related difficulty, interventions addressing mood-like difficulties may be more appropriate. Developmentally informed assessment may therefore guide clinicians toward selecting interventions that target the underlying domain of impairment. Moreover, when irritability emerges early in development, routine screening for co-occurring behavioral, neurodevelopmental, or depressive conditions may be particularly important, given the substantial symptom overlap; approaching irritability within its developmental context may therefore help clarify which additional conditions warrant consideration. Finally, recognizing that irritability may persist into adulthood highlights the importance of identifying and treating clinically significant irritability early before it continues into adulthood, while also acknowledging the need for continued monitoring and support across development.

However, study limitations should be acknowledged. First, attrition was high and likely non-random,^20^^,^^21^ although we attempted to minimize the impact of missing data using full-information maximum likelihood and multiple imputation. Multiple imputation was, however, conducted under the assumption of data missing-at-random, an assumption that cannot be empirically verified. As such, some degree of bias may remain (Supplement 3, available online). Furthermore, our sample is a UK population–based cohort, with limited ethnic diversity; therefore, findings may not generalize to clinically or ethnically diverse populations. Simultaneously, although entropy values were high (Table S3, available online), indicating good classification accuracy, given the relative homogeneity of the ALSPAC cohort and the small size of some profiles, splitting the sample into a discovery and an independent sample to replicate profile stability would be impractical. Replication in independent, and more diverse samples is thus an important area for future research. Moreover, we were unable to investigate irritability before age 7 years, and even after this point, symptoms may have emerged between assessments; therefore, true onset may precede the ages at which irritability elevation was detected in our data. Furthermore, prior work has suggested limited measurement invariance across age in the 3 DAWBA-ODD irritability items,^46^ preventing within-individual assessment of symptom stability/change. In addition, irritability was measured using only 3 items, which, although they align with widely accepted definitions of irritability and with prior work,^2^^,^^3^^,^^8^ may differ in severity and may not capture irritability with optimal precision, especially considering that the scale was not originally developed for irritability measurement. Indeed, irritability was measured within the ODD framework; although irritability has been found to represent a distinct factor within ODD^8^ and despite examining associations with CD as another behavioral condition, the operationalization used in our analyses remains inherently behavioral. Therefore, associations with ODD may have been inflated because of shared measurement context and item overlap. These measurement considerations highlight the need for replication using repeated measures of well-validated instruments specifically designed to measure irritability. Moreover, including both parent- and self-ratings of irritability at age 25 years may have introduced a confound, as they represent different types of information. However, there is evidence of measurement invariance on parent- and self-rated irritability,^46^ and there are theoretical advantages of incorporating multiple perspectives, thereby justifying our choice. Similarly, reporter changes in the DAWBA diagnoses at different ages (informed by the measure available at each age) may have biased the results; for instance, associations between parent-rated irritability and parent-rated diagnoses may differ from those involving parent-rated irritability and self-rated diagnoses. In addition, the covariates were drawn from different developmental timepoints, based on measure availability and developmental relevance; although this approach is common in cohort studies and does not affect class formation (as covariates were examined post hoc),^45^ differences in timing may have introduced heterogeneity, which consequently may have affected interpretation. Moreover, some family history covariates (eg, parental antisocial behavior based on “trouble with the law”) represent broad, self-reported approximations of familial risk; future work incorporating more detailed repeated parental psychiatric data would therefore improve specificity. Furthermore, although the PGS findings were informative, interpretation should be made with caution, as confidence intervals often overlapped across profiles, and as current PGS overall explain only a modest proportion of trait variance; some apparent differences among profiles or mismatches between phenotypic and genetic associations may therefore partly reflect statistical imprecision rather than distinct etiological differences. Finally, separating covariates into behavioral, neurodevelopmental, or depressive categories served as a “heuristic” rather than strict conceptual distinction; some of these covariates may reasonably map onto more than one category. Future work could use approaches such as factor or network analysis to clarify how these covariates cluster, thereby reducing reliance on heuristic groupings.

In conclusion, we applied a developmental framework to investigate behavioral, neurodevelopmental, and depressive types of irritability. We observed elevated rates of behavioral-, neurodevelopmental-, and depressive-like features in most profiles with high irritability, highlighting the heterogeneous and transdiagnostic nature of irritability. We found some evidence consistent with a childhood-onset irritability profile in male participants, which aligned most strongly with neurodevelopmental conditions, and an adolescent-onset irritability profile in female participants, which was consistent primarily with depressive conditions; still, such distinctions have not always been cleanly separated, and our findings remain correlational and not causal. No clearly behavioral irritability type was observed. Overall, our work emphasizes the importance of the developmental context when assessing irritability, with potential diagnostic and treatment implications.

## CRediT authorship contribution statement

**Aikaterini Bekiropoulou:** Writing – original draft, Visualization, Software, Project administration, Investigation, Formal analysis. **Olga Eyre:** Writing – review & editing, Supervision. **Jon Heron:** Writing – review & editing, Supervision, Methodology. **Kate Langley:** Writing – review & editing, Supervision. **Anita Thapar:** Writing – review & editing, Supervision. **Lucy Riglin:** Writing – review & editing, Supervision, Project administration, Methodology, Investigation, Funding acquisition, Conceptualization.
