# Supplementary figures and images for: Using a Developmental Approach to Investigate Behavioral, Neurodevelopmental, and Depressive Irritability Types
Source: JAACAP Open. 2026 Mar 4;4(3):546–62. doi: 10.1016/j.jaacop.2026.02.006 (PMC13221794; doi:10.1016/j.jaacop.2026.02.006)

**
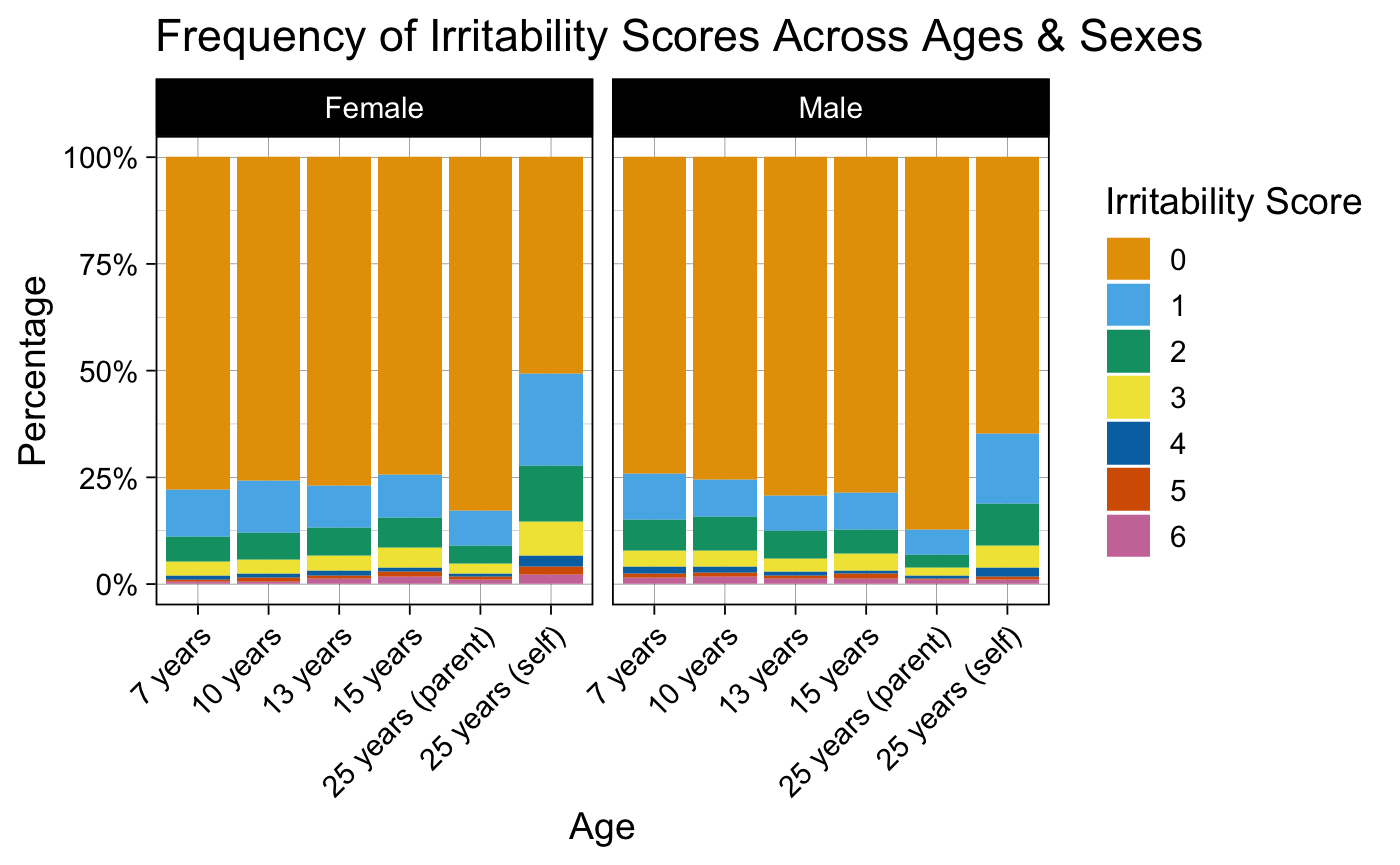
Supplementary Figure 1. Frequency of Irritability Scores Across Ages and Sexes**

Supplement: Supplementary Material [file mmc1.docx]
