# Supplementary material for: Using a Developmental Approach to Investigate Behavioral, Neurodevelopmental, and Depressive Irritability Types
Source: JAACAP Open. 2026 Mar 4;4(3):546–62. doi: 10.1016/j.jaacop.2026.02.006 (PMC13221794; doi:10.1016/j.jaacop.2026.02.006)
Supplement: Supplementary Figure S1 [file mmc2.docx]

**Supplementary Materials**

**Supplement 1: Deviations from the Pre-Registered Protocol**

Our pre-registered protocol stated that we would examine profile sizes (N≥100) along with theoretical interpretability when selecting the optimum number of profiles. On finding a statistically-significant p-value (p<0.0001) in the bootstrapped likelihood ratio test (BLRT), and for reasons of theoretical interpretability, we extended profile testing beyond reaching the minimum class profile initially specified (≥100). Therefore, for the model in the female participants, we selected a solution with a slightly smaller profile size (n=95), as this model refined the adolescent-onset profiles, and adolescent-onset irritability has been identified as theoretically and clinically important in previous work ^1^. This was the only deviation from the pre-registered protocol.

**Supplement 2: Quality Control for Genetic Data**

ALSPAC children were genotyped using the Illumina HumanHap550 quad chip genotyping platforms. As part of standard quality control, gender mismatches, minimal or excessive heterozygosity, disproportionate levels of missingness (>3%), and insufficient sample replication (IBD<0.8) were excluded. Population stratification was assessed using multidimensional scaling analysis and imputed to the Hapmap 2 European descent (CEU), Han Chinese, Japanese, and Yoruba reference populations; individuals with non-European ancestry were removed. Single Nucleotide Polymorphisms (SNPs) with minor allele frequency <1%, call rate <95% or evidence for violations of Hardy-Weinberg equilibrium (P<5E-7) were removed. Cryptic relatedness was measured as the proportion of identity by descent (IBD>0.1); related subjects were removed. Subjects that passed all other quality control thresholds were retained during subsequent phasing and imputation (N=9,115; SNPs=500,527 SNPs). Related individuals were identified using the KING 2.3.1 software ^11^; the individual with the highest genotyping rate from each family was included in the analysis. Principal components analysis was performed in PLINK v1.9 ^12^. More information on the quality control steps undertaken by ALSPAC is described at: <https://www.bristol.ac.uk/media-library/sites/alspac/migrated/documents/gwas-data-generation.pdf>.

**Supplement 3: Sensitivity Analyses**

Within our primary sample (i.e., those included in the longitudinal latent profile analysis: female participants n=5085; male participants n=5028), there were varying levels of missingness for covariates (2.93%-74.14%; Supplementary Table 9). Consistent with our pre-registered protocol, we conducted sensitivity analyses using Multiple Imputation (MI) to impute missing covariate data, a method preferred over alternative approaches (e.g., Inverse Probability Weighting), as it can make use of all available data and impute entire columns of missing information, increasing therefore our study’s statistical power.

Complete-case analyses may bring unbiased estimates when the probability of having complete data depends only on variables included in the analytic model, and not on the unobserved outcome values ^2^. Conversely, analyses based on imputed data are typically expected to be unbiased under the missing-at-random (MAR) assumption, whereby missingness may depend on the observed variables included in the imputation model, but not on the unobserved values of the incomplete variables ^2,3^. Because previous work in ALSPAC has suggested that patterns of attrition and missingness are associated with observed sociodemographic and clinical characteristics ^4–6^, we incorporated these variables as predictors into our imputation model. We also included auxiliary variables, chosen for their conceptual similarity and temporal proximity to the incomplete variables (Supplementary Table 9). Specifically, the variables included in the imputation equations for each covariate included (a) the “best-guess” categorical irritability latent profile, (b) variables collected during pregnancy, showing relatively low missingness and being relevant to factors commonly associated with attrition/missingness ^4–6^, and (c) three assessments of the same or similar construct: two of these were selected as being measured most closely in time to the imputed variable and one in childhood when missingness was less pronounced (Supplementary Table 9). Combining predictors of missingness and auxiliary variables may substantially reduce bias and improve the plausibility of the MAR assumption ^2^. Particularly the inclusion of auxiliary variables may further support the assumptions required for unbiased estimation following imputation by building in additional variables which could not be added to the complete-case estimation without distorting the substantive research question. This justifies the view that, in our context, the assumptions required for unbiased multiple imputation analyses are more plausible than those required for unbiased complete-case analyses.

Multiple imputation analyses were performed in Stata ^7^, and were based on our primary irritability sample, stratified by sex. The use of “best-guess” profile means that classification uncertainty in our latent profile models was not taken into account in these sensitivity analyses. The number of imputations was initially determined as 75, based on the rule of thumb proposed by White et al. ^8^ which recommends setting the number of imputations as approximately equal to the percentage of incomplete cases (2.9%-74.1%; Supplementary Table 9). The number of required imputations was then formally assessed using the “how_many_imputations” command, which is consistent with the recommended 2-stage quadratic rule ^9^. This suggested that, for the model in females, 3-180 imputations were needed depending on the variable, and for males, 3-175. Therefore, 180 and 175 imputed datasets were re-calculated for the female and male participants’ models, respectively; this was deemed sufficient to ensure that the standard errors would remain stable across repeated imputations. The imputed datasets were computed using chained-equations, performed with the “ice” command ^10^; this approach utilized linear (continuous) and logistic (binary) regression-based models, with predictive values estimated based on the observed data (specified in Supplementary Table 9). Results from the imputation models are in Supplementary Tables 10 and 11. Missingness in the irritability assessments are presented by wave, separate for female and male participants, in Supplementary Table 12.

**Supplement 4: Selecting the Number of Latent Profiles**

All models were rerun with double the starts to replicate the best log-likelihood and avoid local maxima. In the female participants, model fit improved from the one- to seven-profile solution according to the loglikelihood, sample size adjusted Bayesian Information Criterion, and BLRT (Supplementary Table 2). The minimum profile size for the six-profile solution was <100 (n=95), so we compared the profiles characterizing the five- and six-profile solutions to examine theoretical interpretability. Compared to the five-profile solution, the six-profile solution included an additional adolescent-onset profile, which has been identified as theoretically and clinically important in previous work ^1^. Cross-tabulation examination of the six- by five-profile solution suggested that most (72.4%) individuals in the adolescence-onset profile were previously classified in the low profile (Supplementary Table 3a), suggesting that the five-profile solution did not capture elevated irritability symptoms in this subgroup efficiently. As model fit also improved from the six- to seven-profile solutions (Supplementary Table 2a), we subsequently examined the profiles characterizing the seven-profile model; in this model, a moderate-stable profile emerged. Cross-tabulation examination of the seven- by six-profile solution suggested that most profiles remained unchanged, while individuals comprising the “moderate-stable” profile primarily came from the “child/adolescent-limited moderate” and the “child-limited” profiles (Supplementary Table 3b). Given the minimal re-classification and considerably small minimum profile size (n=65), the six-profile solution was selected for the female participants.

In the male participants, model fit improved from the one- to seven-profile solution as suggested by the loglikelihood, sample size adjusted Bayesian Information Criterion, and BLRT (Supplementary Table 2b). In the seven-profile solution, a second “high-stable” profile emerged, where both parent- and self-raters suggested relatively high irritability in adulthood. However, the seven-profile solution had a minimum profile size <100 (n=76; Supplementary Table 2b), therefore, similarly with the female models, we used cross-tabulation analysis to investigate theoretical interpretability. This suggested that the general profile structure remained largely unchanged among the seven- and six-profile models, while the newly-emerging profile consisted mainly of those already captured as having high-stable irritability symptoms (Supplementary Table 3c). Considering the small profile size (Supplementary Table 2b), in addition to the minimal profile transitions between the six- and seven-profile solutions, the six-profile model was selected for reasons of parsimony and meaningful interpretation.

**Supplement 5: Code Availability**

The Mplus syntax used to derive the latent profiles of irritability and examine associations with the covariates using the BCH method will be available upon publication at: https://github.com/KaterinaBek/LLPA-BCH.

**References**

1. Riglin L, Eyre O, Thapar AK, et al. Identifying Novel Types of Irritability Using a Developmental Genetic Approach. *American Journal of Psychiatry*. 2019;176(8):635-642. doi:10.1176/appi.ajp.2019.18101134

2. Hughes RA, Heron J, Sterne JAC, Tilling K. Accounting for missing data in statistical analyses: multiple imputation is not always the answer. *Int J Epidemiol*. 2019;48(4):1294-1304. doi:10.1093/ije/dyz032

3. Little R, Rubin D. *Statistical Analysis with Missing Data, Third Edition*. Wiley; 2019. doi:10.1002/9781119482260

4. Boyd A, Golding J, Macleod J, others. Cohort Profile: the `children of the 90s’--the index offspring of the Avon Longitudinal Study of Parents and Children. *Int J Epidemiol*. 2013;42(1):111-127.

5. Fraser A, Macdonald-Wallis C, Tilling K, others. Cohort Profile: the Avon Longitudinal Study of Parents and Children: ALSPAC mothers cohort. *Int J Epidemiol*. 2013;42(1):97-110.

6. Wolke D, Waylen A, Samara M, et al. Selective drop-out in longitudinal studies and non-biased prediction of behaviour disorders. *Br J Psychiatry*. 2009;195(3):249-256. doi:10.1192/bjp.bp.108.053751

7. StataCorp. Stata Statistical Software: Release 19. *College Station, TX: StataCorp LLC*. Preprint posted online 2025.

8. White IR, Royston P, Wood AM. Multiple imputation using chained equations: Issues and guidance for practice. *Stat Med*. 2011;30(4):377-399. doi:10.1002/sim.4067

9. von Hippel PT. How Many Imputations Do You Need? A Two-stage Calculation Using a Quadratic Rule. *Sociol Methods Res*. 2020;49(3):699-718. doi:10.1177/0049124117747303

10. Royston P. Multiple imputation of missing values: Further update of ice, with an emphasis on categorical variables. *Stata Journal*. 2009;9(3):466-477. https://www.stata-journal.com/article.html?article=st0067_4

11. Manichaikul A, Mychaleckyj JC, Rich SS, Daly K, Sale M, Chen WM. Robust relationship inference in genome-wide association studies. *Bioinformatics*. 2010;26(22):2867-2873. doi:10.1093/bioinformatics/btq559

12. Purcell S, Neale B, Todd-Brown K, et al. PLINK: A Tool Set for Whole-Genome Association and Population-Based Linkage Analyses. *The American Journal of Human Genetics*. 2007;81(3):559-575. doi:10.1086/519795

**Table S1. Missingness in Irritability Variables by Assessment Wave**

| Age | Female Participants (n) | Female Participants (%) | Male Participants (n) | Male Participants (%) |
| --- | --- | --- | --- | --- |
| 7 | 1234 | 24.2% | 965 | 19.2% |
| 10 | 1358 | 26.6% | 1238 | 24.6% |
| 13 | 1670 | 32.8% | 1609 | 32% |
| 15 | 2706 | 53.1% | 2826 | 56.2% |
| 25 (parent) | 2782 | 54.6% | 3028 | 60.2% |
| 25 (self) | 2403 | 47.2% | 3678 | 73.2% |

**Table S2. Frequencies of Diagnoses and Clinical Symptoms Across Ages by Sex**

| **Diagnosis/Symptomatology** | **Age** | **Female Participants n (%)** | **Male Participants  n (%)** |
| --- | --- | --- | --- |
| Oppositional Defiant Disorder | 7 years | 71  (1.8%) | 206  (5%) |
|  | 15 years | 83  (3.4%) | 88  (3.9%) |
| Conduct Disorder | 7 years | 15  (0.4%) | 39  (1%) |
|  | 15 years | 39  (1.6%) | 30  (1.3%) |
| Antisocial Behaviors | 25 years | 296  (11.4%) | 237  (18%) |
| Attention-Deficit  Hyperactivity Disorder | 7 years | 30  (0.8%) | 130  (3.2%) |
|  | 15 years | 15  (0.6%) | 28  (1.3%) |
|  | 25 years | 80  (2.9%) | 40  (2.9%) |
| Autism Spectrum Disorder-Like Traits | 7 years | 194  (5.1%) | 392  (9.9%) |
|  | 13 years | 251  (9.1%) | 217  (8.4%) |
|  | 25 years | 331  (12.8%) | 246  (18.9%) |
| Major Depressive Disorder | 7 years | 22  (0.57%) | 30  (0.7%) |
|  | 15 years | 61  (2.2%) | 23  (0.9%) |
|  | 25 years | 298  (11%) | 80  (5.9%) |

**Table S3. Model Fit Indices for Irritability Longitudinal Latent Profiles**

| **Sex** | **Number of Profiles** | **Free Parameters** | **Loglikelihood** | **ssaBIC** | **Entropy** | **Minimum  Profile Size_a_** | **BLRT** |
| --- | --- | --- | --- | --- | --- | --- | --- |
| **Female Participants (a)** | 1 | 12 | -27838.646 | 55741.568 |  | 5085 (100%) |  |
|  | 2 | 19 | -25373.361 | 50848.494 | 0.930 | 442 (9%) | p<0.0001 |
|  | 3 | 26 | -24416.448 | 48972.161 | 0.905 | 186 (4%) | p<0.0001 |
|  | 4 | 33 | -23574.789 | 47326.339 | 0.911 | 116 (2%) | p<0.0001 |
|  | 5 | 40 | -22938.771 | 46091.797 | 0.896 | 121 (2%) | p<0.0001 |
|  | 6_b_ | 47 | -22436.568 | 45124.887 | 0.876 | 95 (2%) | p<0.0001 |
|  | 7 | 54 | -21985.802 | 44260.850 | 0.879 | 65 (1.2%) | p<0.0001 |
| **Male Participants (b)** | 1 | 12 | -26022.361 | 52108.864 |  | 5028 (100%) |  |
|  | 2 | 19 | -23014.268 | 46130.093 | 0.953 | 436 (9%) | p<0.0001 |
|  | 3 | 26 | -21753.593 | 43646.158 | 0.931 | 231 (5%) | p<0.0001 |
|  | 4 | 33 | -21028.014 | 42232.416 | 0.924 | 139 (2.8%) | p<0.0001 |
|  | 5 | 40 | -20346.364 | 40906.532 | 0.907 | 127 (2.6%) | p<0.0001 |
|  | 6_b_ | 47 | -19829.797 | 39910.814 | 0.909 | 117 (2.3%) | p<0.0001 |
|  | 7 | 54 | -19442.206 | 39173.049 | 0.911 | 76 (1.5%) | p<0.0001 |

**Notes:** a. According to most likely profile membership b. Final model selected | ssaBIC= Bayesian Information Criterion sample size adjusted; BLRT=Bootstrapped Likelihood Ratio Test

|  | **Low** | **Child- Limited** | **Child/Adolescent**  **Limited (Moderate)** | **Child/Adolescent-Limited (High)** | **High- Stable** |
| --- | --- | --- | --- | --- | --- |
| **Low** | 4100 (100%) | – | – | – | – |
| **Child-Limited** | – | 297 (95.8%) | – | – | 9  (2.9%) |
| **Child/Adolescent**  **Limited (Moderate)** | – | – | 296  (95.5%) | – | 9  (2.9%) |
| **Child/Adolescent**  **Limited (High)** | 6  (4.8%) | 5  (4%) | – | 98  (78.4%) | 14  (11.2%) |
| **High-Stable** | – | – | – | 6  (6.3%) | 87  (91.6%) |
| **Adolescent-Onset_a_** | 105 (72.4%) | – | 14  (9.7%) | 16  (11%) | 6  (4.1%) |

**Table S4a. Cross-Tabulation Analysis for the 6-profile by 5-profile models (Female Participants)**

**Notes:** a. New profile **|** Cells marked “ – ” indicate n<5; this may include n=0

**Table S4b. Cross-Tabulation Analysis for the 7-class by 6-class models (Female Participants)**

|  | **Low** | **Child- Limited** | **Child/Adolescent**  **Limited (Moderate)** | **Child/Adolescent-Limited (High)** | **High- Stable** | **Adolescent-Onset** |
| --- | --- | --- | --- | --- | --- | --- |
| **Low** | 4095 (99.6%) | – | – | – | – | 15  (0.4%) |
| **Child-Limited** | – | 272  (100%) | – | – | – | – |
| **Child/Adolescent**  **Limited (Moderate)** | – | – | 251  (99.6%) | – | – | – |
| **Child/Adolescent**  **Limited (High)** | – | – | 6  (5.8%) | 97  (93.3%) | – | – |
| **High-Stable** | – | – | – | – | 64 (98.5%) | – |
| **Adolescent-**  **Onset** | – | – | – | – | – | 122  (94.6%) |
| **Moderate- Stable_a_** | – | 35 (23.3%) | 50  (33.3%) | 27  (18%) | 30  (20%) | 8  (5.3%) |

**Notes:** a. New profile **|** Cells marked “ – ” indicate n<5; this may include n=0

**Table S4c. Cross-Tabulation Analysis for the 7-class by 6-class models (Male Participants)**

|  | **Low** | **Child-Limited** | **Child/Adolescent**  **Limited (Moderate)** | **Child/Adolescent-Limited (High)** | **High-**  **Stable** | **Fluctuating** |
| --- | --- | --- | --- | --- | --- | --- |
| **Low** | 4054 (100%) | – | – | – | – | – |
| **Child-Limited** | 9  (3.6%) | 238 (94.8%) | – | – | – | – |
| **Child/Adolescent**  **Limited (Moderate)** | – | – | 296  (100%) | – | – | – |
| **Child/Adolescent**  **Limited (High)** | – | 5  (4%) | 8  (6.5%) | 107  (86.3%) | – | – |
| **High-Stable** | – | – | 6  (6.5%) | 13  (14%) | 69  (74.2%) | – |
| **Fluctuating** | 6  (4.5%) | – | – | – | – | 127  (94.8%) |
| **High-Stable/both raters*** | – | – | – | 27  (35.5%) | 46  (60.5%) | – |

**Notes:** a. New profile where both parental- and self-reports suggest relatively elevated irritability at age 2 **|** Cells marked “ – ” indicate n<5; this may include n=0

**Table S5. Means and Standard Errors Across Ages by Latent Profile and Sex**

| **Female  Participants (a)** | **Age** | **Low** | **Child-Limited** | **Child/Adolescent-Limited (Moderate)** | **Child/Adolescent-Limited (High)** | **High-Stable** | **Adolescence-Onset** |
| --- | --- | --- | --- | --- | --- | --- | --- |
|  | 7 years | 0.11 (0.01) | 2.64 (0.04) | 0.59 (0.04) | 1.06 (0.07) | 3.91 (0.08) | 0.22 (0.05) |
|  | 10 years | 0.20 (0.02) | 1.25 (0.06) | 0.86 (0.05) | 2.22 (0.10) | 3.88 (0.10) | 0.58 (0.10) |
|  | 13 years | 0.09 (0.01) | 0.36 (0.03) | 2.39 (0.03) | 5.19 (0.06) | 3.31 (0.06) | 0.38 (0.06) |
|  | 15 years | 0.23 (0.02) | 0.92 (0.09) | 1.48 (0.08) | 3.44 (0.16) | 3.72 (0.16) | 2.34 (0.15) |
|  | 25 years (parent) | 0.11 (0.01) | 0.32 (0.05) | 0.49 (0.05) | 1.21 (0.09) | 4.36 (0.10) | 3.62 (0.09) |
|  | 25 years (self) | 0.85 (0.04) | 1.04 (0.12) | 1.63 (0.13) | 2.17 (0.28) | 2.68 (0.28) | 2.60 (0.45) |
| **Male Participants (b)** | **Age** | **Low** | **Child-Limited** | **Child/Adolescent-Limited (Moderate)** | **Child/Adolescent-Limited (High)** | **High-Stable** | **Fluctuating** |
|  | 7 years | 0.17 (0.01) | 3.18 (0.07) | 0.82 (0.05) | 2.45 (0.08) | 4.85 (0.09) | 0.62 (0.08) |
|  | 10 years | 0.16 (0.02) | 1.52 (0.08) | 1.13 (0.05) | 3.46 (0.10) | 4.93 (0.11) | 2.42 (0.10) |
|  | 13 years | 0.08 (0.01) | 0.19 (0.03) | 2.33 (0.02) | 5.41 (0.05) | 2.75 (0.05) | 0.43 (0.05) |
|  | 15 years | 0.20 (0.02) | 0.67 (0.09) | 1.34 (0.08) | 3.93 (0.18) | 3.39 (0.18) | 2.38 (0.17) |
|  | 25 years (parent) | 0.08 (0.02) | 0.29 (0.06) | 0.41 (0.06) | 1.86 (0.11) | 1.25 (0.12) | 4.19 (0.09) |
|  | 25 years (self) | 0.58 (0.04) | 0.53 (0.15) | 1.18 (0.14) | 1.26 (0.32) | 1.85 (0.31) | 2.40 (0.24) |

**Notes:** Values are presented as “Mean (Standard Error)”; Means and standard errors were rounded to 2 decimal places

|  | **Low** | **Child-Limited** | |  | **Child/Adolescent- Limited (Moderate)** | |  | **Child/Adolescent- Limited (High)** | |  | **High-Stable** | |  | **Adolescent-Onset** | |  |
| --- | --- | --- | --- | --- | --- | --- | --- | --- | --- | --- | --- | --- | --- | --- | --- | --- |
| **Variables** | **Prevalence (SE)** | **Prevalence (SE)** | **Chi-square (p-value)**_a_ | | **Prevalence (SE)** | **Chi-square  (p-value)** | | **Prevalence (SE)** | **Chi-square  (p-value)** | | **Prevalence (SE)** | **Chi-square  (p-value)** | | **Prevalence (SE)** | **Chi-square  (p-value)** | |
| **Behavioral** |  |  |  | |  |  | |  |  | |  |  | |  |  | |
| Oppositional Defiant Disorder 7y | – | **14.4%** (2.2%) | 43.287  (<0.001) | | – | 0.500 (0.480) | | 4.7%  (3%) | 2.686 (0.101) | | **36.7%** (6.1%) | 36.436 (<0.001) | | – | 0.170 (0.680) | |
| Oppositional Defiant Disorder 15y | – | 2.1%  (1.5%) | 3.050 (0.081) | | **8.5%**  (2.2%) | 15.951  (<0.001) | | **48.6%**  (8.9%) | 30.469  (<0.001) | | **42.5%**  (7.6%) | 31.932  (<0.001) | | **18.2%**  (5.3%) | 11.924 (0.001) | |
| Conduct Disorder 7y | – | **2.6%**  (1%) | 6.749  (0.009) | | – | 0.906  (0.341) | | – | 0.471 (0.493) | | **7.8%**  (3.4%) | 5.298 (0.021) | | – | 5.803 (0.016) | |
| Conduct Disorder 15y | – | – | 0.945 (0.331) | | **4.3%**  (1.6%) | 7.290 (0.007) | | **18.4%**  (6.5%) | 8.076 (0.004) | | **13.7%**  (5.3%) | 6.707 (0.010) | | **11.1%**  (4.2%) | 6.854 (0.009) | |
| Antisocial  Behaviors 25y | 9.3%  (0.7%) | 9.5%  (2.6%) | 0.011 (0.916) | | **17.7%**  (3.3%) | 6.085 (0.014) | | **23.8%**  (7.1%) | 4.111 (0.043) | | **29.7%**  (7.8%) | 6.858 (0.009) | | **26.6%**  (5.7%) | 8.555 (0.003) | |
| **Neurodevelopmental** |  |  |  | |  |  | |  |  | |  |  | |  |  | |
| ADHD 7y | 0.2%  (0.1%) | **3.3%**  (1.1%) | 7.300 (0.007) | | – | 0.109 (0.741) | | – | 0.083 (0.774) | | **13.6%**  (4.3%) | 9.843 (0.002) | | – | 0.051 (0.821) | |
| ADHD 15y | – | – | 1.808 (0.179) | | – | 0.214 (0.644) | | 4%  (3.5%) | 1.372 (0.241) | | **15.9%**  (5.6%) | 8.227 (0.004) | | 5.5%  (3%) | 3.170 (0.075) | |
| ADHD 25y | 2.1%  (0.4%) | 3.5%  (1.6%) | 0.703 (0.402) | | 1.8%  (1.1%) | 0.081 (0.775) | | 5.3%  (3.9%) | 0.665 (0.415) | | **20.9%**  (6.7%) | 7.844 (0.005) | | **10.4%**  (3.9%) | 4.209 (0.040) | |
| ASD-Like Traits 7y | 1.7%  (0.3%) | **22.8%**  (2.7%) | 57.665 (<0.001) | | **5%**  (1.5%) | 4.626 (0.031) | | **16.8%**  (4.8%) | 9.746 (0.002) | | **52.6%**  (6.6%) | 60.047 (<0.001) | | – | 0.060 (0.807) | |
| ASD-Like Traits 13y | 3.5%  (0.5%) | **11.8%**  (2.5%) | 9.991 (0.002) | | **19.4%**  (2.8%) | 28.815  (<0.001) | | **67.2%**  (8.9%) | 51.503  (<0.001) | | **58.6%**  (7.9%) | 48.287  (<0.001) | | **30.9%**  (6.6%) | 16.542  (<0.001) | |
| ASD-Like Traits 25y | 10.4%  (0.7%) | 14.2%  (3.1%) | 1.398 (0.237) | | 11.5%  (2.7%) | 0.144 (0.704) | | **40.6%**  (8.3%) | 12.979  (<0.001) | | **42.1%**  (8.3%) | 14.386  (<0.001) | | **27.9%**  (5.8%) | 8.583 (0.003) | |
| Low IQ | 4.7%  (0.5%) | 5.8%  (1.7%) | 0.373 (0.541) | | **8.9%**  (1.9%) | 4.406 (0.036) | | 13.1%  (4.5%) | 3.350 (0.067) | | **19.6%**  (5%) | 8.867 (0.003) | | 11.4%  (4.3%) | 2.274 (0.132) | |
| Special Education Needs | 13.3%  (0.9%) | 19.8%  (3.9%) | 2.667 (0.102) | | 16.1%  (3.2%) | 0.726 (0.394) | | **36.8%**  (7.9%) | 8.714 (0.003) | | **32.1%**  (7.3%) | 6.523 (0.011) | | 22.2%  (7.2%) | 1.472 (0.225) | |
| **Depressive** |  |  |  | |  |  | |  |  | |  |  | |  |  | |
| Major Depressive Disorder 7y | – | **4.8%**  (1.3%) | 12.356  (<0.001) | | – | 0.439 (0.508) | | – | 12.668  (<0.001) | | – | 1.726 (0.189) | | – | 6.276 (0.012) | |
| Major Depressive Disorder 15y | 1.9%  (0.3%) | 4.6%  (1.7%) | 2.532 (0.112) | | 2.5%  (1.2%) | 0.302 (0.583) | | – | 0.102 (0.750) | | 5.8%  (3.3%) | 1.364 (0.243) | | – | 0.017 (0.896) | |
| Major Depressive Disorder 25y | 8.2%  (0.7%) | 9.8%  (2.6%) | 0.325 (0.569) | | **15.5%**  (3%) | 5.189 (0.023) | | **29.4%**  (7.4%) | 7.980 (0.005) | | **27.4%**  (7.3%) | 6.805 (0.009) | | **38.2%**  (6.2%) | 21.781 (<0.001) | |

**Table S6a. Prevalence of Clinical, Educational, and Cognitive Covariates by Irritability Latent Profile, and Comparison to the Low Profile (Female Participants)
Notes:** a. original p-values; statistically significant values are in bold | Chi-square compared to the low profile (df=1) | Cells marked “ – ” indicate n<5; this may include 0% | ADHD= Attention-Deficit Hyperactivity Disorder; ASD=Autism Spectrum Disorder; SE=Standard Errors

**Table S6b.** **Prevalence of Clinical, Educational, and Cognitive Covariates by Irritability Latent Profile, and comparison to the low profile (Male Participants)**

|  | **Low** | **Child-Limited** | |  | **Child/Adolescent- Limited (Moderate)** | |  | **Child/Adolescent- Limited (High)** | |  | **High-Stable** | |  | **Fluctuating** | |  |
| --- | --- | --- | --- | --- | --- | --- | --- | --- | --- | --- | --- | --- | --- | --- | --- | --- |
| **Variables** | **Prevalence (SE)** | **Prevalence (SE)** | **Chi-square (p-value)**_a_ | | **Prevalence (SE)** | **Chi-square  (p-value)** | | **Prevalence (SE)** | **Chi-square  (p-value)** | | **Prevalence (SE)** | **Chi-square  (p-value)** | | **Prevalence (SE)** | **Chi-square  (p-value)** | |
| **Behavioral** |  |  |  | |  |  | |  |  | |  |  | |  |  | |
| Oppositional Defiant Disorder 7y | – | **31%**  (3.5%) | 74.692  (<0.001) | | **4.8%**  (1.4%) | 10.471 (0.001) | | **24.4%**  (4.8%) | 25.365  (<0.001) | | **80.4%**  (4.8%) | 285.192 (<0.001) | | 4.5%  (2.9%) | 2.408 (0.121) | |
| Oppositional Defiant Disorder 15y | – | – | 0.173 0.677 | | **12.4%**  (2.7%) | 19.489  (<0.001) | | **89.4%**  (18.9%) | 22.449  (<0.001) | | **54.5%**  (8.9%) | 37.661  (<0.001) | | **27.4%**  (6.6%) | 16.912  (<0.001) | |
| Conduct Disorder 7y | – | **3.4%**  (1.5%) | 5.265 (0.022) | | – | 1.231 (0.267) | | **11.5%**  (3.4%) | 11.534 (0.001) | | **15.7%**  (4.1%) | 14.631  (<0.001) | | – | 0.075 (0.784) | |
| Conduct Disorder 15y | – | – | 0.024 (0.876) | | **3%**  (1.4%) | 3.898 (0.048) | | **33.4%**  (13%) | 6.619 (0.010) | | **11.3%**  (5.6%) | 4.018 (0.045) | | **15.1%**  (5.2%) | 8.254 (0.004) | |
| Antisocial  Behaviors 25y | 15.8%  (1.2%) | 13.3%  (5.2%) | 0.228 (0.633) | | **31.8%**  (5.6%) | 7.648 (0.006) | | 27%  (23.9%) | 0.219 (0.640) | | 35.7%  (12.4%) | 2.570 (0.109) | | **37.6%**  (8.9%) | 5.837 (0.016) | |
| **Neurodevelopmental** |  |  |  | |  |  | |  |  | |  |  | |  |  | |
| ADHD 7y | 0.4%  (0.2%) | **13.2%**  (2.6%) | 23.029  (<0.001) | | **4.3%**  (1.3%) | 7.854 (0.005) | | **23%**  (4.6%) | 23.837  (<0.001) | | **33.4%**  (5.2%) | 39.692  (<0.001) | | **8.1%**  (3.2%) | 5.591 (0.018) | |
| ADHD 15y | – | 3.3%  (2.1%) | 2.158 (0.142) | | 1.8%  (1.1%) | 2.007 (0.157) | | **33.7%**  (13%) | 6.640 (0.010) | | **14.3%**  (6.1%) | 5.299 (0.021) | | 7.1%  (3.8%) | 3.319 (0.068) | |
| ADHD 25y | 2%  (0.4%) | – | 0.417 (0.519) | | 4.9%  (2.5%) | 1.197 (0.274) | | 19.4%  (17%) | 1.039 (0.308) | | **23.2%**  (10.4%) | 4.112 (0.043) | | 12.5%  (6.2%) | 2.809 (0.094) | |
| ASD-Like Traits 7y | 2.2%  (0.4%) | **52.1%**  (4%) | 152.209  (<0.001) | | **20.7%**  (2.7%) | 45.618  (<0.001) | | **42.8%**  (5.8%) | 48.894  (<0.001) | | **68.7%**  (5.4%) | 152.782  (<0.001) | | **16.6%**  (4.7%) | 9.332 (0.002) | |
| ASD-Like Traits 13y | 3.3%  (0.5%) | **21.3%**  (4.4%) | 15.948  (<0.001) | | **23.3%**  (3.2%) | 37.294  (<0.001) | | **98.3%**  (16.1%) | 34.951  (<0.001) | | **48.1%**  (8.7%) | 26.424  (<0.001) | | **21.6%**  (6%) | 9.282 (0.002) | |
| ASD-Like Traits 25y | 18.1%  (1.2%) | 12.7%  (5.4%) | 0.925 (0.336) | | 19.7%  (5%) | 0.101 (0.750) | | 42.7%  (24.9%) | 0.982 (0.322) | | 39.8%  (12.2%) | 3.157 (0.076) | | 33.5%  (8.9%) | 2.912 (0.088) | |
| Low IQ | 5.9%  (0.5%) | 8.1%  (2.6%) | 0.743 (0.389) | | 8.6%  (1.9%) | 1.975 (0.160) | | **36.4%**  (7.5%) | 16.747  (<0.001) | | **15.9%**  (4.7%) | 4.563 (0.033) | | 8.7%  (3.6%) | 0.614 (0.433) | |
| Special Education Needs | 27.8%  (1.1%) | **48.8%**  (5.7%) | 12.821  (<0.001) | | 29.5%  (3.8%) | 0.172 (0.678) | | **72%**  (8%) | 30.136  (<0.001) | | **60.3%**  (7.6%) | 17.870  (<0.001) | | 38.9%  (7.4%) | 2.141 (0.143) | |
| **Depressive** |  |  |  | |  |  | |  |  | |  |  | |  |  | |
| Major Depressive Disorder 7y | – | **3.8%**  (1.5%) | 6.222 (0.013) | | – | 1.162 (0.281) | | 4.5%  (2.3%) | 3.777 (0.052) | | **12%**  (3.6%) | 10.835 (0.001) | | – | 17.433  (<0.001) | |
| Major Depressive Disorder 15y | 0.5%  (0.2%) | – | 0.001 (0.981) | | – | 0.404 (0.525) | | 11.5%  (7.1%) | 2.372 (0.124) | | 5.1%  (3.7%) | 1.548 (0.213) | | 4.9%  (2.9%) | 2.166 (0.141) | |
| Major Depressive Disorder 25y | 4.7%  (0.7%) | 7.9%  (4%) | 0.624 (0.430) | | 6.3%  (3%) | 0.258 (0.612) | | – | 0.040 (0.842) | | 11.4%  (7.9%) | 0.717 (0.397) | | **30.1%**  (8.3%) | 9.180 (0.002) | |

**Notes:** a. original p-values; statistically significant values are in bold | Chi-square compared to the low profile (df=1) | Cells marked “ – ” indicate n<5; this may include 0% | ADHD= Attention-Deficit Hyperactivity Disorder; ASD=Autism Spectrum Disorder; SE=Standard Errors

**Table S7a.** **Means and Prevalence of Genetic, Family History and Environmental Covariates by Irritability Latent Profile and Comparison to the Low Profile (Female Participants)**

|  | **Low** | **Child-Limited** | |  | **Child/Adolescent- Limited (Moderate)** | |  | **Child/Adolescent- Limited (High)** | | |  | **High-Stable** | |  | **Adolescent-Onset** | | |  |
| --- | --- | --- | --- | --- | --- | --- | --- | --- | --- | --- | --- | --- | --- | --- | --- | --- | --- | --- |
| **Variables** | **Prevalence/Mean (SE)** | **Prevalence/ Mean (SE)** | **Chi-square (p-value)**_a_ | | **Prevalence/Mean (SE)** | **Chi-square  (p-value)** | | **Prevalence/Mean (SE)** | | **Chi-square  (p-value)** | | **Prevalence/**  **Mean (SE)** | **Chi-square  (p-value)** | | **Prevalence/Mean (SE)** | | **Chi-square  (p-value)** | |
| **Behavioral** |  |  |  | |  |  | |  |  | | |  |  | |  |  | | |
| Maternal Antisocial Behaviors | 7.1%  (0.6%) | **14.1%**  (2.5%) | 7.190 (0.007) | | 10.4%  (2%) | 2.421 (0.120) | | **21.9%**  (5.7%) | 6.567 (0.010) | | | **19.1%**  (5.4%) | 4.897 (0.027) | | 8.6%  (3.9%) | 0.138 (0.710) | | |
| Paternal Antisocial Behaviors | 28.4%  (1.4%) | 35.5%  (5.2%) | 1.676 (0.195) | | 38.4%  (5%) | 3.504 (0.061) | | 60.1%  (20.9%) | 2.294 (0.130) | | | 21.9%  (9.4%) | 0.473 (0.492) | | 39.3%  (8.4%) | 1.555 (0.212) | | |
| Parental Conflict | 9.1%  (0.6%) | **14.6%**  (2.4%) | 4.693 (0.030) | | **15.9%**  (2.5%) | 6.662 (0.010) | | **24.5%**  (5.9%) | 6.822 (0.009) | | | **26.3%**  (6.1%) | 7.976 (0.005) | | 19.9%  (5.5%) | 3.710 (0.054) | | |
| **Neuro-**  **developmental** |  |  |  | |  |  | |  |  | | |  |  | |  |  | | |
| ADHD PGS_b_ | -0.076 (0.020) | -0.002 (0.076) | 0.869 (0.351) | | **0.080** (0.071) | 4.271 (0.039) | | **0.337** (0.150) | 7.398 (0.007) | | | 0.157 (0.149) | 2.409 (0.121) | | **0.333** (0.128) | 9.648 (0.002) | | |
| ASD PGS_b_ | -0.027 (0.021) | 0.060 (0.076) | 1.178 (0.278) | | 0.096 (0.069) | 2.834 (0.092) | | 0.145 (0.127) | 1.792 (0.181) | | | 0.211 (0.153) | 2.392 (0.122) | | 0.195 (0.143) | 2.295 (0.130) | | |
| Preterm Birth | 4.5%  (0.4%) | 2.6%  (1%) | 2.742 (0.098) | | 3.2%  (1.1%) | 1.222 (0.269) | | – | 0.288 (0.591) | | | 5.9%  (2.6%) | 0.290 (0.590) | | 4.6%  (2.3%) | 0.002 (0.963) | | |
| Low Birth Weight | 3.4%  (0.3%) | 3.4%  (1.2%) | 0.002 (0.965) | | 5.8%  (1.4%) | 2.387 (0.122) | | 4.3%  (2.3%) | 0.144 (0.705) | | | – | 0.000 (0.990) | | 4.7%  (2.4%) | 0.252 (0.616) | | |
| Low Apgar Score | 0.4%  (0.1%) | – | 0.056 (0.814) | | – | 0.047 (0.828) | | – | 0.457 (0.499) | | | – | 9.140 (0.003) | | – | 10.108 (0.001) | | |
| **Depressive** |  |  |  | |  |  | |  |  | | |  |  | |  |  | | |
| Major Depressive Disorder PGS_b_ | -0.092 (0.020) | 0.033 (0.072) | 2.726 (0.099) | | **0.100** (0.066) | 7.259 (0.007) | | **0.266** (0.133) | 7.059 (0.008) | | | **0.190** (0.133) | 4.384 (0.036) | | **0.673** (0.150) | 24.525  (<0.001) | | |
| Maternal Depression | 6%  (0.4%) | **11.3%**  (2%) | 6.401 (0.011) | | **12.7%**  (2.1%) | 9.551 (0.002) | | **16.2%**  (4.1%) | 6.007 (0.014) | | | 9.6%  (3.4%) | 1.056 (0.304) | | **15.6%**  (4.1%) | 5.276 (0.022) | | |
| Paternal Depression | 5.8%  (0.5%) | 6%  (1.8%) | 0.018 (0.894) | | 6.7%  (1.9%) | 0.222 (0.637) | | 13.8%  (5%) | 2.517 (0.113) | | | – | 3.688 (0.055) | | 3.5%  (2.5%) | 0.768 (0.381) | | |
| Stressful Life  Events (16y)_b_ | 5.740 (0.106) | 6.319 (0.281) | 3.573 (0.059) | | 6.299 (0.376) | 1.958 (0.162) | | **9.469** (1.127) | 10.818 (0.001) | | | 7.052 (0.844) | 2.381 (0.123) | | **7.732** (0.769) | 6.317 (0.012) | | |
| Stressful Life  Events (25y)_b_ | 5.911 (0.118) | 5.821 (0.453) | 0.036 (0.850) | | **7.205** (0.540) | 5.270 (0.022) | | 5.113 (0.647) | 1.467 (0.226) | | | 7.534 (0.956) | 2.839 (0.092) | | **8.493** (0.857) | 8.532 (0.003) | | |

**Notes:** a. original p-values; statistically significant values are in bold | b. Means | Chi-square tests compared to the low profile as reference (df=1) | Cells marked “ – ” indicate n<5; this may include 0% | ADHD=Attention Deficit Hyperactivity Disorder; ASD=Autism Spectrum Disorder; PGS=Polygenic Scores; SE=Standard Errors

|  | **Low** | **Child-Limited** | |  | **Child/Adolescent- Limited (Moderate)** | |  | **Child/Adolescent- Limited (High)** | | |  | **High-Stable** | |  | **Fluctuating** | | |  |
| --- | --- | --- | --- | --- | --- | --- | --- | --- | --- | --- | --- | --- | --- | --- | --- | --- | --- | --- |
| **Variables** | **Prevalence/Mean (SE)** | **Prevalence/Mean (SE)** | **Chi-square (p-value)**_a_ | | **Prevalence/Mean (SE)** | **Chi-square  (p-value)** | | **Prevalence/Mean (SE)** | | **Chi-square  (p-value)** | | **Prevalence/**  **Mean (SE)** | **Chi-square  (p-value)** | | **Prevalence/Mean (SE)** | | **Chi-square  (p-value)** | |
| **Behavioral** |  |  |  | |  |  | |  |  | | |  |  | |  |  | | |
| Maternal Antisocial Behaviors | 6.6%  (0.5%) | 12.2%  (2.9%) | 3.424 (0.064) | | 8.3%  (1.9%) | 0.723 (0.395) | | **21.3%**  (7.1%) | 4.321 (0.038) | | | 14.8%  (4.5%) | 3.259 (0.071) | | 15.4%  (4.5%) | 3.619 (0.057) | | |
| Paternal Antisocial Behaviors | 28%  (1.3%) | 27.2%  (5.9%) | 0.019 (0.889) | | 34.4%  (4.9%) | 1.546 (0.214) | | 60%  (17.3%) | 3.389 (0.066) | | | 21.6%  (9.6%) | 0.445 (0.505) | | 37.3%  (12.8%) | 0.506 (0.477) | | |
| Parental Conflict | 8.7%  (0.5%) | 12.6%  (2.9%) | 1.703 (0.192) | | 11.7%  (2.2%) | 1.812 (0.178) | | 17.2%  (4.8%) | 3.211 (0.073) | | | 15.3%  (4.3%) | 2.322 (0.128) | | **20.1%**  (4.5%) | 6.180 (0.013) | | |
| **Neuro-**  **developmental** |  |  |  | |  |  | |  |  | | |  |  | |  |  | | |
| ADHD PGS_b_ | -0.085 (0.019) | -0.001 (0.085) | 0.887 (0.346) | | **0.059** (0.069) | 3.855 (0.050) | | 0.189 (0.154) | 3.090 (0.079) | | | **0.259** (0.102) | 11.017 (0.001) | | **0.250** (0.105) | 9.642 (0.002) | | |
| ASD PGS_b_ | 0.001 (0.019) | 0.061 (0.089) | 0.428 (0.513) | | -0.059 (0.065) | 0.760 (0.383) | | 0.094 (0.142) | 0.416 (0.519) | | | 0.036 (0.120) | 0.086 (0.769) | | 0.080 (0.111) | 0.477 (0.490) | | |
| Preterm Birth | 4.7%  (0.4%) | 7.4%  (2%) | 1.717 (0.190) | | 5%  (1.3%) | 0.047 (0.829) | | 6.3%  (2.6%) | 0.341 (0.560) | | | 10%  (3%) | 3.176 (0.075) | | 6.9%  (2.5%) | 0.736 (0.391) | | |
| Low Birth Weight | 3.5%  (0.3%) | 5.3%  (1.7%) | 1.072 (0.301) | | 2.7%  (1%) | 0.563 (0.453) | | 5.3%  (2.4%) | 0.487 (0.485) | | | 6.6%  (2.5%) | 1.502 (0.220) | | 3.8%  (1.9%) | 0.020 (0.887) | | |
| Low Apgar Score | 0.6%  (0.2%) | – | 0.448 (0.503) | | – | 0.008 (0.931) | | – | 0.106 (0.745) | | | – | 2.215 (0.137) | | – | 16.574  (<0.001) | | |
| **Depressive** |  |  |  | |  |  | |  |  | | |  |  | |  |  | | |
| Major Depressive Disorder PGS_b_ | -0.082 (0.019) | -0.020 (0.080) | 0.550 (0.458) | | 0.052 (0.072) | 3.132 (0.077) | | **0.249** (0.146) | 4.995 (0.025) | | | **0.224** (0.110) | 7.451 (0.006) | | **0.280** (0.113) | 9.780 (0.002) | | |
| Maternal Depression | 6.6%  (0.4%) | **13.5%**  (2.7%) | 6.301 (0.012) | | 9.2%  (1.8%) | 1.939 (0.164) | | **20.2%**  (4.2%) | 10.210 (0.001) | | | **15.8%**  (3.7%) | 6.095 (0.014) | | 5.8%  (2.6%) | 0.102 (0.749) | | |
| Paternal Depression | 4.4%  (0.4%) | **10.4%**  (2.7%) | 4.559 (0.033) | | 5.3%  (1.6%) | 0.291 (0.589) | | 8.2%  (3.9%) | 0.957 (0.328) | | | **13.9%**  (4%) | 5.655 (0.017) | | 6.3%  (3.3%) | 0.349 (0.555) | | |
| Stressful Life  Events (16y)_b_ | 4.235 (0.096) | 4.761 (0.489) | 1.086 (0.297) | | **5.436** (0.442) | 6.830 (0.009) | | 7.260 (2.622) | 1.329 (0.249) | | | **6.436** (0.975) | 5.052 (0.025) | | 4.926 (1.008) | 0.461 (0.497) | | |
| Stressful Life  Events (25y)_b_ | 4.931 (0.145) | 4.386 (0.527) | 0.966 (0.326) | | 6.071 (0.582) | 3.513 (0.061) | | 5.898 (2.680) | 0.130 (0.719) | | | 4.139 (1.105) | 0.505 (0.477) | | 6.356 (0.976) | 2.048 (0.152) | | |

**Table S7b. Means and Prevalence of Genetic, Family History and Environmental Covariates by Irritability Latent Profile and Comparison to the Low Profile (Male Participants)**

**Notes:** a. original p-values; statistically significant values are in bold | b. Means | Chi-square tests compared to the low profile as reference (df=1) | Cells marked “ – ” indicate n<5; this may include 0% | ADHD=Attention Deficit Hyperactivity Disorder; ASD=Autism Spectrum Disorder; PGS=Polygenic Scores; SE=Standard Errors

**Table S8. High-Stable Profile vs Other Irritability Profiles on Neurodevelopmental Covariates (Male Participants)**

|  | **Child-Limited** | **Child/Adolescent- Limited Moderate** | **Child/Adolescent- Limited High** | **Fluctuating** |
| --- | --- | --- | --- | --- |
| ADHD 7y | 11.655 **(0.007/0.001)** | 28.853  **(0.007/<0.001)** | 2.102  (0.276/0.147) | 16.686  **(0.007/<0.001)** |
| ASD-like Traits 7y | 6.058 (0.061/**0.014**) | 63.163  **(0.007/<0.001)** | 10.281  **(0.007/0.001)** | 52.698  **(0.007/<0.001)** |
| ADHD 15y | 2.774 (0.238/0.096) | 3.978  (0.141/**0.046**) | 1.763  (0.319/0.184) | 0.953  (0.475/0.329) |
| ASD-like Traits 13y | 7.405 **(0.040/0.007)** | 7.096  **(0.042/0.008)** | 7.350  **(0.040/0.007)** | 6.216  (0.061/**0.013**) |
| ADHD 25y | 4.388 (0.134/**0.036**) | 2.879  (0.234/0.090) | 0.035  (0.904/0.852) | 0.769  (0.507/0.380) |
| ASD-like Traits 25y | 4.065 (0.141/**0.044**) | 2.288  (0.276/0.130) | 0.011  (0.934/0.916) | 0.169  (0.795/0.681) |
| Special Education Needs | 1.430 (0.377/0.232) | 12.915  **(0.007/<0.001)** | 1.074  (0.446/0.300) | 4.030  (0.141/**0.045**) |
| Low IQ | 2.065 (0.276/0.151) | 2.035  (0.276/0.154) | 5.241  (0.088/**0.022**) | 1.453  (0.377/0.228) |
| ADHD PGS | 3.774 (0.150/0.052) | 2.570  (0.258/0.109) | 0.137  (0.804/0.711) | 0.003  (0.955/0.955) |
| ASD PGS | 0.026 (0.906/0.871) | 0.485  (0.602/0.486) | 0.090  (0.846/0.765) | 0.068  (0.860/0.794) |
| Preterm Birth | 0.535 (0.588/0.464) | 2.342  (0.276/0.126) | 0.861  (0.498/0.354) | 0.641  (0.551/0.424) |
| Low Birth Weight | 0.165 (0.795/0.684) | 2.060  (0.276/0.151) | 0.161  (0.795/0.688) | 0.773  (0.507/0.379) |
| Low Apgar – 5minutes | 1.126 (0.442/0.289) | 2.136  (0.276/0.144) | 1.244  (0.418/0.265) | 3.236  (0.197/0.072) |

**Notes:** Results are presented as chi-square (q-value/p-value); False Discovery Rate-adjusted for multiple testing (52 tests) | Prevalences are shown in Tables 3b and 4b of the main manuscript | Statistically-significant q-/p-values are in bold | ADHD=Attention Deficit Hyperactivity Disorder; ASD=Autism Spectrum Disorder; PGS=Polygenic Scores

|  | **Child-Limited** | **Child/Adolescent- Limited Moderate** | **Child/Adolescent- Limited High** | **High-Stable** |
| --- | --- | --- | --- | --- |
| MDD 7y | 14.477 **(0.006/<0.001)** | 2.066  (0.333/0.151) | 2.862  (0.241/0.091) | 2.014 (0.333/0.156) |
| MDD 15y | 0.901 (0.502/0.342) | 0.030  (0.891/0.863) | 0.102  (0.799/0.749) | 0.831 (0.504/0.362) |
| MDD 25y | 17.383 **(0.006/<0.001)** | 10.454  **(0.006/0.001)** | 0.772  (0.505/0.379) | 1.218 (0.455/0.270) |
| MDD PGS | 14.593 **(0.006/<0.001)** | 11.891  **(0.006/0.001)** | 3.872  (0.174/**0.049**) | 5.557 (0.072/**0.018**) |
| Maternal Depression | 0.892 (0.502/0.345) | 0.388  (0.632/0.533) | 0.008  (0.930/0.930) | 1.237 (0.455/0.266) |
| Paternal Depression | 0.678 (0.525/0.410) | 1.026  (0.498/0.311) | 3.197  (0.237/0.074) | 0.297 (0.647/0.586) |
| Stressful Life Events 16y | 2.951 (0.241/0.086) | 2.736  (0.241/0.098) | 1.551  (0.401/0.213) | 0.340 (0.640/0.560) |
| Stressful Life Events 25y | 7.501 **(0.027/0.006)** | 1.565  (0.401/0.211) | 9.408  **(0.011/0.002)** | 0.536 (0.571/0.464) |

**Table S9. Adolescent-Onset Profile vs Other Irritability Profiles on Depressive Covariates (Female Participants)**

**Notes:** Results are presented as chi-square (q-value/p-value); False Discovery Rate-adjusted for multiple testing (52 tests) | Prevalences are shown in Tables 3a and 4a of the main manuscript | Statistically-significant q-/p-values are in bold | ADHD=Attention Deficit Hyperactivity Disorder; ASD=Autism Spectrum Disorder; PGS=Polygenic Scores

**Table S10a. Variables Included in the Imputation Model (Clinical, Educational, Cognitive)**

| **Incomplete Covariate** | **Missing %**  **(female participants)** | **Missing %**  **(male participants)** | **Model** | **Variables Used for Imputation** |
| --- | --- | --- | --- | --- |
| ODD 7y | 2.9% | 18.2% | Logistic | Irritability profile_a_, Predictors of missingness_b_, SDQ-conduct subscale (ages 4, 6)_c_, DAWBA-ODD_d_ diagnosis (age 10) |
| ODD 15y | 52% | 55.5% | Logistic | Irritability profile, Predictors of missingness, SDQ-conduct subscale (ages 4, 16), DAWBA-ODD diagnosis (age 13) |
| Conduct Disorder 7y | 23.6% | 18.8% | Logistic | Irritability profile, Predictors of missingness, SDQ-conduct subscale (ages 4, 6), DAWBA-Conduct disorder diagnosis (age 10) |
| Conduct Disorder 15y | 52% | 55.5% | Logistic | Irritability profile, Predictors of missingness, SDQ-conduct subscale (ages 4, 16), DAWBA-Conduct disorder diagnosis (age 13) |
| Antisocial Behaviors 25y | 48.7% | 73.8% | Logistic | Irritability profile, Predictors of missingness, SDQ-conduct subscale (ages 4, 25), Antisocial behaviors (self-rated; age 23) |
| ADHD 7y | 22.73% | 18.1% | Logistic | Irritability profile, Predictors of missingness, SDQ-hyperactivity subscale (ages 4, 6), DAWBA-ADHD diagnosis (age 10) |
| ADHD 15y | 51.9% | 55.4% | Logistic | Irritability profile, Predictors of missingness, SDQ-hyperactivity subscale (ages 4, 16), DAWBA-ADHD diagnosis (age 13) |
| ADHD 25y | 46.5% | 72.6% | Logistic | Irritability profile, Predictors of missingness, SDQ-hyperactivity subscale (ages 4, 25), BAARS-IV_e_ (age 25) |
| ASD-Like Traits 7y | 25.6% | 20.9% | Logistic | Irritability profile, Predictors of missingness, Child autism diagnosis (parent-rated, linked), SCDC_f_ (ages 13, 17) |
| ASD-Like Traits 13y | 45.5% | 48.8% | Logistic | Irritability profile, Predictors of missingness, Child autism diagnosis (parent-rated, linked), SCDC (ages 7, 17) |
| ASD-Like Traits 25y | 49% | 74.1% | Logistic | Irritability profile, Predictors of missingness, Adult autism diagnosis (self-rated), SCDC (ages 7, 25) |
| MDD 7y | 23.7% | 19.4% | Logistic | Irritability profile, Predictors of missingness, SMFQ (ages 9, 11)_g_, DAWBA-MDD diagnosis (age 10) |
| MDD 15y | 46% | 50.8% | Logistic | Irritability profile, Predictors of missingness, SMFQ (age 16), DAWBA-MDD diagnosis (ages 7, 13) |
| MDD 25y | 46.8% | 73% | Logistic | Irritability profile, Predictors of missingness, SMFQ (ages 21, 23), DAWBA-MDD diagnosis (age 7) |
| Low IQ 8y | 31.3% | 32.4% | Logistic | Irritability profile, Predictors of missingness, Special education needs (parent-rated: age 7; teacher-rated: age 12), IQ (age 15) |
| Special Education Needs 12y | 54.5% | 54.9% | Logistic | Irritability profile, Predictors of missingness, Special education needs (parent-rated: age 7), IQ (ages 8, 15) |

**Notes:** a. Irritability profiles (across sexes: low, child-limited, child/adolescent-limited moderate, child/adolescent-limited high, high stable; female sample: adolescent-onset; male sample : fluctuating); b. Predictors of missingness: maternal age at birth, maternal social class by occupation (birth), maternal education (birth), parity, crowding index; c. SDQ: Strengths and Difficulties Questionnaire (parent-rated), d. DAWBA: Development and Well-Being Assessment (parent-rated: ODD, Conduct Disorder, ADHD, MDD ages 7, 10, 13; self-rated: MDD ages 15, 25); e. BAARS-IV: Barkley Adult ADHD Rating Scale-IV (parent-rated); f. SCDC: Social and Communication Disorder Checklist (parent-rated); g. SMFQ: Short Mood and Feelings Questionnaire (parent-rated: age 9; self-rated: ages 11, 16, 21, 23) | ADHD=Attention Deficit Hyperactivity Disorder; ASD=Autism-Spectrum Disorder; MDD= Major Depressive Disorder; ODD=Oppositional Defiant Disorder

**Table S10b. Variables Included in the Imputation Model (Genetic, Family History, Environmental)**

| **Incomplete Covariate** | **Missing %**  **(female participants)** | **Missing %**  **(male participants)** | **Model** | **Variables Used for Imputation** |
| --- | --- | --- | --- | --- |
| Maternal Lifetime Trouble with the Law 12y | 33.8% | 32.8% | Logistic | Irritability profile_a_, Predictors of missingness_b_, Maternal trouble with the law (8-weeks postpartum), Maternal Trouble with Police <17 years (32-weeks gestation), Parental Conflict (age 6) |
| Paternal Lifetime Trouble with the Law 12y | 70.9% | 70.2% | Logistic | Irritability profile, predictors of missingness, Paternal Trouble with the Law (8-weeks postpartum), Parental Conflict (age 6), Domestic Violence (father towards mother; 18-weeks gestation) |
| Parental Conflict 6y | 30.7% | 26.2% | Logistic | Irritability profile, predictors of missingness, Aggression Score (12-weeks gestation), Affection Score (12-weeks gestation), Domestic Violence (father towards mother; 18-weeks gestation) |
| ADHD PGS | 49.6% | 28.6% | Linear | Irritability profile, Predictors of missingness, SDQ-hyperactivity subscale (age 4)_c_, DAWBA-ADHD_d_ diagnosis (ages 15, 25) |
| Autism Spectrum Disorder PGS | 49.6% | 28.6% | Linear | Irritability profile, Predictors of missingness, SCDC_e_ (ages 7, 13), Autism Quotient (self-rated, age 25) |
| Preterm Birth | 6.3% | 4.3% | Logistic | Irritability profile, Predictors of missingness, Birth weight, Apgar score (1 and 5 minutes) |
| Low Birth Weight | 7.4% | 5.2% | Logistic | Irritability profile, Predictors of missingness, Gestational length, Apgar score (1 and 5 minutes) |
| Low Apgar Score  (5 minutes) | 44.3% | 44% | Logistic | Irritability profile, Predictors of missingness, Birth weight, Gestational length, Apgar score (1 minute) |
| MDD PGS | 49.6% | 28.6% | Linear | Irritability profile, predictors of missingness, DAWBA-MDD diagnosis (ages 7, 15, 25) |
| Maternal Lifetime Depression  (12-weeks gestation) | 10.1% | 8.2% | Logistic | Irritability profile, predictors of missingness, Maternal depression (ages 3: since child 18-months, 8 years: lifetime), Maternal EPDS_f_ (8-weeks postpartum) |
| Paternal Lifetime Depression  (12-weeks gestation) | 36.58% | 35.8% | Logistic | Irritability profile, predictors of missingness, Paternal depression (ages 3: since child 18-months, 8 years: lifetime), Paternal EPDS (8-weeks postpartum) |
| Stressful Life Events 16y | 46.4% | 62.7% | Linear | Irritability profile, predictors of missingness, Stressful life events (ages 4, 17, 25) |
| Stressful Life Events 25y | 49.8% | 74.1% | Linear | Irritability profile, predictors of missingness, Stressful life events (ages 4, 16, 17) |

**Notes:** a. Irritability profiles (across sexes: low, child-limited, child/adolescent-limited moderate, child/adolescent-limited high, high stable; female sample: adolescent-onset; male sample: fluctuating); b. Predictors of missingness: maternal age at birth, maternal social class by occupation (birth), maternal education (birth), parity, crowding index; c. SDQ: Strengths and Difficulties Questionnaire (parent-rated), d. DAWBA: Development and Well-Being Assessment (parent-rated: ADHD, MDD ages 7; self-rated: MDD ages 15, 25); e. SCDC: Social and Communication Disorder Checklist (parent-rated); f. EPDS: Edinburgh Postnatal Depression Scale | ADHD=Attention Deficit Hyperactivity Disorder; MDD=Major Depressive Disorder; PGS=Polygenic Scores

|  | **Low** | **Child-Limited** | **Child/Adolescent-Limited Moderate** | **Child/Adolescent-Limited High** | **High-Stable** | **Adolescent-Onset** |
| --- | --- | --- | --- | --- | --- | --- |
| **Variables** | **Prevalence (SE)** | **Prevalence (SE)** | **Prevalence (SE)** | **Prevalence (SE)** | **Prevalence (SE)** | **Prevalence (SE)** |
| **Behavioral** |  |  |  |  |  |  |
| Oppositional Defiant Disorder 7y | 0.1%  (0.2%) | 12.5%  (0.7%) | 0.3%  (0.8%) | 6.6%  (1.8%) | 30.6%  (2.6%) | 0.9%  (1.1%) |
| Oppositional Defiant Disorder 15y | 1.2%  (0.4%) | 4.5%  (1.4%) | 9%  (1.5%) | 36.5%  (4.1%) | 41.2%  (4.1%) | 11.1%  (1.7%) |
| Conduct Disorder7y | 0.1%  (0.1%) | 2.3%  (0.5%) | 0.6%  (0.5%) | 3.6%  (1.5%) | 10.6%  (2.5%) | 0.2%  (0.7%) |
| Conduct Disorder15y | 0.9%  (0.3%) | 2.4%  (1.1%) | 4.6%  (1%) | 15.5%  (2.9%) | 14.2%  (3%) | 6.9%  (1.4%) |
| Antisocial Behaviors 25y | 10.8%  (0.7%) | 13%  (2.5%) | 16.5%  (2.5%) | 22.4%  (4.6%) | 29.4%  (5.5%) | 21.3%  (3.4%) |
| **Neurodevelopmental** |  |  |  |  |  |  |
| ADHD 7y | 0.4%  (0.2%) | 3%  (0.6%) | 0.6%  (0.6%) | 3.5%  (1.6%) | 14.3%  (3%) | 0.9%  (0.8%) |
| ADHD 15y | 0.2%  (0.2%) | 0.6%  (0.7%) | 0.7%  (0.7%) | 5.4%  (2.2%) | 14.8%  (2.9%) | 2.9%  (0.8%) |
| ADHD 25y | 2.7%  (0.4%) | 4.4%  (1.4%) | 3.4%  (1.4%) | 7.7%  (2.8%) | 16.3%  (3.7%) | 8.8%  (2%) |
| ASD-Like Traits 7y | 2.7%  (0.4%) | 20.2%  (1.4%) | 7.2%  (1.6%) | 21.3%  (3.1%) | 49.3%  (3.6%) | 4.4%  (2%) |
| ASD-Like Traits 13y | 4.8%  (0.5%) | 12.6%  (1.9%) | 20.4%  (2%) | 51.9%  (4%) | 55.8%  (4.6%) | 20.6%  (2.8%) |
| ASD-Like Traits 25y | 12.3%  (0.8%) | 17.1%  (2.6%) | 15.3%  (2.7%) | 34.6%  (4.6%) | 44%  (5.8%) | 26.5%  (3.7%) |
| Low IQ | 6.2%  (0.5%) | 1.7%  (1.8%) | 3.9%  (1.8%) | 9.8%  (3.2%) | 16.2%  (3.6%) | 4.2%  (2.5%) |
| Special Education Needs | 45.5%  (1.9%) | 53.5%  (3.7%) | 51.2%  (4%) | 67.6%  (5.5%) | 64.5%  (6.2%) | 12.5%  (5.3%) |
| **Depressive** |  |  |  |  |  |  |
| Major Depressive Disorder 7y | 0.3%  (0.1%) | 4.3%  (0.5%) | 0.6%  (0.6%) | 1%  (1.2%) | 4.2%  (1.7%) | 0.3%  (0.8%) |
| Major Depressive Disorder 15y | 2.3%  (0.4%) | 4.4%  (1.3%) | 3.1%  (1.2%) | 3.5 %  (2.3%) | 5.8%  (2.6%) | 2.9%  (1.7%) |
| Major Depressive Disorder 25y | 9.8%  (0.6%) | 12.7%  (2.3%) | 15.1%  (2.4%) | 25%  (4.3%) | 27.9%  (5.1%) | 27.7%  (3.4%) |

**Table S11a. Prevalence of Clinical, Educational, and Cognitive Covariates by Irritability Profile Compared to the Low Profile (Female Participants; Imputation)**

**Notes:** The respective models using original (non-imputed) data are in Table 3a | ADHD=Attention Deficit Hyperactivity Disorder; ASD=Autism Spectrum Disorder; SE=Standard Errors

**Table S11b. Prevalence of Clinical, Educational, and Cognitive Covariates by Irritability Profile Compared to the Low Profile (Male Participants; Imputation)**

|  | **Low** | **Child-Limited** | **Child/Adolescent-Limited Moderate** | **Child/Adolescent-Limited High** | **High-Stable** | **Fluctuating** |
| --- | --- | --- | --- | --- | --- | --- |
| **Variables** | **Prevalence (SE)** | **Prevalence (SE)** | **Prevalence (SE)** | **Prevalence (SE)** | **Prevalence (SE)** | **Prevalence (SE)** |
| **Behavioral** |  |  |  |  |  |  |
| Oppositional Defiant Disorder 7y | 0.6%  (0.2%) | 26.7%  (1.1%) | 4.6%  (1%) | 19.9%  (1.5%) | 60%  (1.8%) | 6%  (1.7%) |
| Oppositional Defiant Disorder 15y | 1.6%  (3.9%) | 7%  (2%) | 12%  (1.8%) | 26.3%  (3.5%) | 52.4%  (4%) | 28.7%  (4%) |
| Conduct Disorder 7y | 0.2%  (0.2%) | 3.4%  (0.7%) | 0.8%  (0.6%) | 8.6%  (1%) | 12.6%  (1.3%) | 1.8%  (1.2%) |
| Conduct Disorder 15y | 0.7%  (0.3%) | 2%  (1.2%) | 2.7%  (1%) | 10.7%  (2%) | 20%  (3.8%) | 13.3%  (3.8%) |
| Antisocial Behaviors 25y | 17.4%  (1.3%) | 20.4%  (3.5%) | 29.3%  (3.8%) | 16.2%  (4.7%) | 35.2%  (6.7%) | 41.9%  (6.7%) |
| **Neurodevelopmental** |  |  |  |  |  |  |
| ADHD 7y | 1%  (0.3%) | 12.2%  (1.4%) | 4.3%  (1.1%) | 17.8%  (1.6%) | 29.1%  (2.1%) | 10.5%  (2%) |
| ADHD 15y | 0.7%  (0.3%) | 4.3%  (1.3%) | 2.8%  (1.1%) | 10.7%  (2.3%) | 18.8%  (3.5%) | 7.8%  (2.6%) |
| ADHD 25y | 3.2%  (0.6%) | 22.3%  (1.9%) | 6.4%  (1.9%) | 12%  (1.9%) | 18.2%  (4.4%) | 14.9%  (4.6%) |
| ASD-Like Traits 7y | 4.3%  (0.5%) | 44.3%  (1.9%) | 21.3%  (1.9%) | 35.5%  (2.6%) | 62.8%  (3.4%) | 27.3%  (3.5%) |
| ASD-Like Traits 13y | 4.5%  (0.5%) | 20.7%  (2.4%) | 22.5%  (2%) | 36.3%  (3.6%) | 56.8%  (4.9%) | 32.9%  (4%) |
| ASD-Like Traits 25y | 19.4%  (1.2%) | 21.5%  (4.1%) | 23.2%  (3.6%) | 28.8%  (6%) | 35.7%  (7.2%) | 33.4%  (6.6%) |
| Low IQ | 7.2%  (0.5%) | 9.3%  (2.1%) | 9.3%  (1.8%) | 21.3%  (3%) | 14.9%  (3.3%) | 10.8%  (3%) |
| Special Education Needs | 50.3%  (1.7%) | 62%  (4.3%) | 51%  (4.1%) | 70.4%  (6.2%) | 69.8%  (7.4%) | 60.8%  (7.2%) |
| **Depressive** |  |  |  |  |  |  |
| Major Depressive Disorder 7y | 0.2%  (0.1%) | 3.3%  (0.6%) | 0.8%  (0.6%) | 3.5%  (0.8%) | 10.1%  (1.4%) | 1.1%  (1.2%) |
| Major Depressive Disorder 15y | 0.9%  (0.3%) | 1.5%  (1%) | 1.8%  (1%) | 3.3%  (1.5%) | 6%  (3.3%) | 5.1%  (2%) |
| Major Depressive Disorder 25y | 5.4%  (0.7%) | 9.6%  (2.6%) | 11.3%  (2.5%) | 6.7%  (3.1%) | 21.1%  (5.5%) | 26%  (5.3%) |

**Notes:** The respective models using original (non-imputed) data are in Table 3b | ADHD=Attention Deficit Hyperactivity Disorder; ASD=Autism Spectrum Disorder; SE=Standard Errors

|  | **Low** | **Child-Limited** | **Child/Adolescent-Limited Moderate** | **Child/Adolescent-Limited High** | **High-Stable** | **Adolescent-Onset** |
| --- | --- | --- | --- | --- | --- | --- |
| **Variables** | **Prevalence/ Mean (SE)** | **Prevalence/ Mean (SE)** | **Prevalence/ Mean (SE)** | **Prevalence/ Mean (SE)** | **Prevalence/ Mean (SE)** | **Prevalence/ Mean (SE)** |
| **Behavioral** |  |  |  |  |  |  |
| Maternal Antisocial Behaviors | 8%  (0.5%) | 13.3%  (1.9%) | 10.9%  (1.9%) | 17%  (3.2%) | 19.8%  (4.1%) | 9.7%  (2.8%) |
| Paternal Antisocial Behaviors | 31.6%  (1.4%) | 34.1%  (3.7%) | 36%  (4%) | 41.8%  (6.8%) | 39%  (8%) | 34.9%  (5.2%) |
| Parental Conflict | 10.6%  (0.6%) | 14.9%  (2.1%) | 17.6%  (2.4%) | 24.6%  (3.9%) | 28.2%  (4.7%) | 17.6%  (3.2%) |
| **Neurodevelopmental** |  |  |  |  |  |  |
| ADHD PGS_a_ | -0.046  (0.019) | 0.010  (0.066) | 0.085  (0.066) | 0.262  (0.108) | 0.222  (0.127) | 0.174  (0.097) |
| ASD PGS_a_ | -0.014  (0.019) | 0.050  (0.069) | 0.095  (0.069) | 0.143  (0.109) | 0.187  (0.130) | 0.094  (0.099) |
| Preterm Birth | 4.6%  (0.3%) | 3.5%  (1.3%) | 3.7%  (1.3%) | 3.7%  (2%) | 5.9%  (2.3%) | 4.6%  (1.8%) |
| Low Birth Weight | 3.7%  (0.3%) | 4.1%  (1.2%) | 6.1%  (1.2%) | 4.5%  (1.9%) | 3.6%  (2.1%) | 4.7%  (1.7%) |
| Low Apgar Score | 0.3%  (0.1%) | 0.4%  (0.4%) | 0.5%  (0.4%) | 1.2%  (0.8%) | 0.4%  (1.1%) | 0.3%  (0.6%) |
| **Depressive** |  |  |  |  |  |  |
| Major Depressive Disorder PGS_a_ | -0.052  (0.183) | 0.040  (0.066) | 0.098  (0.066) | 0.214  (0.106) | 0.208  (0.126) | 0.357  (0.099) |
| Maternal Depression | 6.6%  (0.4%) | 11.3%  (1.6%) | 12.5%  (2.7%) | 15.1%  (2.6%) | 11.9%  (3.2%) | 13.5%  (2.4%) |
| Paternal Depression | 6.6%  (0.5%) | 7.6%  (1.8%) | 7.4%  (1.8%) | 12.5%  (3.2%) | 6%  (3.6%) | 6%  (2.5%) |
| Stressful Life Events (16y)_a_ | 5.941  (0.099) | 6.393  (0.314) | 6.535  (0.334) | 7.818  (0.568) | 7.409  (0.639) | 6.960  (0.462) |
| Stressful Life Events (25y)_a_ | 6.107  (0.108) | 6.139  (0.380) | 6.755  (0.400) | 6.153  (0.628) | 6.847  (0.758) | 7.320  (0.521) |

**Table S12a. Means/Prevalence of Genetic, Family History and Environmental Covariates by Irritability Profile Compared to the Low Profile (Female Participants; Imputation)**

**Notes:** The respective models using original (non-imputed) data are in Table 4a | a. Means | ADHD=Attention Deficit Hyperactivity Disorder; ASD=Autism Spectrum Disorder;
PGS=Polygenic Scores; SE=Standard Errors

**Table S12b. Means/Prevalence of Genetic, Family History and Environmental Covariates by Irritability Profile Compared to the Low Profile (Male Participants; Imputation)**

|  | **Low** | **Child-Limited** | **Child/Adolescent-Limited Moderate** | **Child/Adolescent-Limited High** | **High-Stable** | **Fluctuating** |
| --- | --- | --- | --- | --- | --- | --- |
| **Variable** | **Prevalence/ Mean (SE)** | **Prevalence/ Mean (SE)** | **Prevalence/ Mean (SE)** | **Prevalence/ Mean (SE)** | **Prevalence/ Mean (SE)** | **Prevalence/ Mean (SE)** |
| **Behavioral** |  |  |  |  |  |  |
| Maternal Antisocial Behaviors | 7.3%  (0.5%) | 12.1%  (2.2%) | 8.8%  (1.9%) | 13.2%  (2.8%) | 13.4%  (3.2%) | 14%  (3.1%) |
| Paternal Antisocial Behaviors | 30%  (1.3%) | 30.4%  (4.1%) | 33.2%  (4.1%) | 34.2%  (5.5%) | 32.9%  (6.6%) | 35.7%  (7.3%) |
| Parental Conflict | 9.7%  (0.6%) | 13.7%  (2.3%) | 13.1%  (2.1%) | 15.6%  (3%) | 17.4%  (3.6%) | 18.5%  (3.3%) |
| **Neurodevelopmental** |  |  |  |  |  |  |
| ADHD PGS_a_ | -0.068  (0.018) | 0.020  (0.741) | 0.063  (0.065) | 0.103  (0.962) | 0.247  (0.105) | 0.233  (0.101) |
| ASD PGS_a_ | <0.001  (0.018) | 0.051  (0.073) | <0.001  (0.065) | 0.060  (0.096) | 0.368  (0.105) | 0.042  (0.072) |
| Preterm Birth | 5.3%  (2%) | 7.4%  (1.6%) | 5.4%  (1.4%) | 6.3%  (2%) | 10.1%  (2.3%) | 7.3%  (2.1%) |
| Low Birth Weight | 3.9%  (0.3%) | 5.6%  (1.4%) | 2.9%  (1.2%) | 5.1%  (1.7%) | 6.6%  (2%) | 4.2%  (1.8%) |
| Low Apgar Score | 0.5%  (0.2%) | 1%  (0.6%) | 0.6%  (0.5%) | 0.8%  (0.7%) | 2.9%  (1%) | 0.5%  (1.1%) |
| **Depressive** |  |  |  |  |  |  |
| Major Depressive Disorder PGS_a_ | -0.064  (0.019) | 0.008  (0.074) | 0.058  (0.067) | 0.120  (0.961) | 0.219  (0.105) | 0.267  (0.100) |
| Maternal Depression | 6.9%  (0.4%) | 13%  (1.8%) | 9%  (1.6%) | 17.4%  (2.3%) | 7.8%  (2.7%) | 7%  (2.5%) |
| Paternal Depression | 4.8%  (0.4%) | 8.8%  (1.7%) | 5.9%  (1.6%) | 8.3%  (2.4%) | 14.3  (2.9%) | 8.6%  (2.9%) |
| Stressful Life Events (16y)_a_ | 4.425  (0.101) | 5.016  (0.330) | 5.275  (0.311) | 4.83  (0.469) | 6.06  (0.565) | 5.738  (0.557) |
| Stressful Life Events (25y)_a_ | 5.050  (0.135) | 5.227  (0.422) | 5.587  (0.415) | 5.228  (0.562) | 5.634  (0.727) | 6.023  (0.727) |

**Notes:** a. Means | The respective models using original (non-imputed) data are in Table 4b | ADHD=Attention Deficit Hyperactivity Disorder; ASD=Autism Spectrum
Disorder; PGS=Polygenic Scores; SE=Standard Errors
